# Diversification and independent domestication of Asian and European pears

**DOI:** 10.1186/s13059-018-1452-y

**Published:** 2018-06-11

**Authors:** Jun Wu, Yingtao Wang, Jiabao Xu, Schuyler S. Korban, Zhangjun Fei, Shutian Tao, Ray Ming, Shuaishuai Tai, Awais M. Khan, Joseph D. Postman, Chao Gu, Hao Yin, Danman Zheng, Kaijie Qi, Yong Li, Runze Wang, Cecilia H. Deng, Satish Kumar, David Chagné, Xiaolong Li, Juyou Wu, Xiaosan Huang, Huping Zhang, Zhihua Xie, Xiao Li, Mingyue Zhang, Yanhong Li, Zhen Yue, Xiaodong Fang, Jiaming Li, Leiting Li, Cong Jin, Mengfan Qin, Jiaying Zhang, Xiao Wu, Yaqi Ke, Jian Wang, Huanmimg Yang, Shaoling Zhang

**Affiliations:** 1 Centre of Pear Engineering Technology Research, State Key Laboratory of Crop Genetics and Germplasm Enhancement, Nanjing Agricultural University, Nanjing, 210095 China; 2 Hebei Academy of Agriculture and Forestry Sciences, Shijiazhuang Fruit Tree Research Institute, Shijiazhuang, 050061 China; 3 BGI Genomics, BGI-Shenzhen, Shenzhen, 518083 China; 4 University of Illinois at Urbana-Champaign, Urbana, IL 61801 USA; 5 Plant Pathology and Plant-Microbe Section, Cornell University, Geneva, NY 14853 USA; 6 USDA-ARS, Boyce Thompson Institute, Ithaca, NY 14853 USA; 7 Fujian Agriculture and Forestry University, Fuzhou, 350002 China; 8 USDA-ARS National Clonal Germplasm Repository, Corvallis, OR 97333 USA; 9 The New Zealand Institute for Plant & Food Research Limited, Auckland, New Zealand; 10 James D. Watson Institute of Genome Sciences, Hangzhou, 310058 China

**Keywords:** Pear (*Pyrus*), Re-sequencing genomes, Origin and evolution, Independent domestication, Fruit-related traits, Self-incompatibility

## Abstract

**Background:**

Pear (*Pyrus*) is a globally grown fruit, with thousands of cultivars in five domesticated species and dozens of wild species. However, little is known about the evolutionary history of these pear species and what has contributed to the distinct phenotypic traits between Asian pears and European pears.

**Results:**

We report the genome resequencing of 113 pear accessions from worldwide collections, representing both cultivated and wild pear species. Based on 18,302,883 identified SNPs, we conduct phylogenetics, population structure, gene flow, and selective sweep analyses. Furthermore, we propose a model for the divergence, dissemination, and independent domestication of Asian and European pears in which pear, after originating in southwest China and then being disseminated throughout central Asia, has eventually spread to western Asia, and then on to Europe. We find evidence for rapid evolution and balancing selection for S-RNase genes that have contributed to the maintenance of self-incompatibility, thus promoting outcrossing and accounting for pear genome diversity across the Eurasian continent. In addition, separate selective sweep signatures between Asian pears and European pears, combined with co-localized QTLs and differentially expressed genes, underline distinct phenotypic fruit traits, including flesh texture, sugar, acidity, aroma, and stone cells.

**Conclusions:**

This study provides further clarification of the evolutionary history of pear along with independent domestication of Asian and European pears. Furthermore, it provides substantive and valuable genomic resources that will significantly advance pear improvement and molecular breeding efforts.

**Electronic supplementary material:**

The online version of this article (10.1186/s13059-018-1452-y) contains supplementary material, which is available to authorized users.

## Background

Pear (*Pyrus*), one of the most economically important temperate fruit tree species, with an annual worldwide production of ~ 18 million tons (2015, FAOSTAT), belongs to the subtribe Malinae of the Amygdaloideae subfamily within Rosaceae [1]. The genus *Pyrus* includes at least 22 recognized species [2], with more than 5000 accessions maintained worldwide. These accessions display wide morphological and physiological variability, as well as broad adaptations to wide agro-ecological ranges. As a self-incompatible flowering plant, pear is an obligate outcrosser. It is important to note that hybridization in pear occurred not only intraspecies but also interspecies, despite its wide geographic distribution. Although many pear groups are deemed as different species, they are in fact rather similar to subpopulations based primarily on their distinguishable phenotypes. Therefore, it is likely that inter-‘species’ hybridizations and genetic admixtures must have occurred among pear groups without reproductive barriers [3]. Nevertheless, they have long been widely recognized and deemed as “species” in pear research studies [4, 5].

The ancient *Pyrus* lineage probably arose during the Tertiary period, between 65 to 55 million years ago (MYA), in the mountainous regions of southwestern China [5]. Subsequently, it was dispersed across mountainous ranges both eastward and westward. This oriental and occidental geographical distribution of pear led to the respective development of Asian and European pears [6]. The earliest cultivation of Asian pears can be traced back to about 3300 years ago [7], with commercial orchards known to have existed for more than 2000 years in China [8]. Similarly, European pears have been cultivated for more than 3000 years, with distinct named cultivars recorded as early as 300 B.C. [9].

*Pyrus communis*, the predominant cultivated species of European pear, bears typical pear-shaped fruits with soft and smooth flesh, few stone cells, along with strong aroma and flavor. The major cultivated species in Asia, including *P. pyrifolia*, *P. bretschneideri*, *P. sinkiangensis*, and *P. ussuriensis*, bear round-shaped fruits with crisp flesh, high sugar content, low acid content, minimal aroma, and mild flavor. The genetic variations and domestication processes responsible for these observed phenotypic differences in fruit trait characters between European and Asian pears are not well understood.

Indeed, these wide genetic variations present in pear accessions, belonging to various *Pyrus* species, have made it quite difficult to identify relationships among pear germplasm collections. Consequently, current available pear DNA sequence data are inadequate to delineate clear population-level relationships among various pear species [10–12]. In the past two decades, whole-genome sequencing tools have revolutionized the field of life sciences as they have provided unprecedented new means and opportunities to explore and understand genetic variation, evolution, and domestication processes of agricultural crops [13]. Owing to its self-incompatibility and long generation cycle, among other factors, genetic and molecular analyses of pear have been rather challenging and slow. However, recent completion of whole-genome sequencing of ‘Dangshansuli’, an Asian pear [14], and “Bartlett”, a European pear [15], has yielded new knowledge, including characterization of the genomic structure, chromosome evolution, and patterns of genetic variation related to important agricultural traits.

In this study, we conducted population-level analysis of genetic variation of pears based on the resequencing of genomes of a diverse group of 57 wild and 56 cultivated *Pyrus* accessions from wide geographical regions. A total of 18,302,883 genome-wide SNPs were identified and used in multiple analyses. Findings were then used to propose a model to explain divergence, dissemination, and domestication of Asian and European pears. Of particular note, analysis of the evolutionary rate and balancing selection of *S*-locus genes highlights the impact of self-incompatibility on the genetic diversity of pear, which likely had a strong influence on gene flow and observed genetic variations in *Pyrus*. Furthermore, selective sweeps associated with agriculturally important genes were detected in cultivated Asian and European pears. In addition to evolutionary and functional genomics insights, this study provides an unprecedented amount of genomic data that will almost certainly enable important advances in modern pear improvement and molecular breeding programs.

## Results and discussion

### Sequencing and mapping of pear accessions

Genomes of 113 *Pyrus* accessions, including 63 Asian (31 cultivated and 32 wild) and 50 European (25 cultivated and 25 wild) pears, were sequenced (Additional file 1: Figure S1). A total of 661 Gb of high-quality cleaned sequence data were generated, with an average of 5.85 Gb per accession (equivalent to approximately 11× coverage of the ~ 527-Mb pear genome). These sequences were aligned to the Asian pear genome ‘Dangshansuli’ [14], with an average mapping rate of 61.39% (Additional file 2), and a total of 18,302,883 SNPs were identified with ~ 90 SNPs per kb (Table 1, Additional file 1: Figure S2). It is important to note that considerable care was taken in selecting ‘Dangshansuli’ over the European pear genome Bartlett as the reference genome for read alignment in this study. This was a necessary step as there were two published pear genomes under consideration. However, a pseudo-alignment between these two genomes revealed high divergence, which would result in a low mapping rate for cross-species alignment. Specifically, we first looked at the quality parameters of the two published pear genomes, including the contig N50, the scaffold N50, and the scaffold size values, as well as the scaffold-to-chromosome anchoring ratios. Results revealed that the quality of the ‘Dangshansuli’ genome was higher than that of the Bartlett genome (Additional file 1: Table S1). This also suggested that no matter which genome was selected, it is unlikely that the mapping rates for divergent samples would be improved (Additional file 1: Note 1). It was ultimately decided that the high-quality ‘Dangshansuli’ Asian pear genome would be the reference in this study.

**Table 1:** Summary of genetic diversity in different pear groups

Pear accessions—groups (number of samples)	Effective site	SNP number	ϴ_w_	ϴ_π_	*Tajima’s D*
All (113)	203,042,855	18,302,883	1.56E-02	5.50E-03	− 0.81
Asian (63)	232,249,059	14,501,253	1.20E-02	5.24E-03	− 0.83
European (50)	173,858,920	6,945,796	8.07E-03	3.71E-03	− 0.86
Cultivated (56)	214,202,044	10,902,511	1.01E-02	5.64E-03	− 0.72
Wild (57)	188,713,516	13,540,936	1.42E-02	5.15E-03	− 0.95
Cultivated Asian (31)	243,314,658	8,441,743	7.76E-03	4.76E-03	− 0.80
Wild Asian (32)	215,884,040	10,510,280	1.08E-02	5.21E-03	− 0.97
Cultivated European (25)	191,912,058	4,220,232	5.21E-03	3.57E-03	− 0.78
Wild European (25)	155,765,734	4,894,247	7.38E-03	3.53E-03	− 1.09

Among all identified SNPs, a total of 14.1% were located in coding regions: 7.7% were non-synonymous and 6.4% were synonymous, with a non-synonymous/synonymous ratio of 1.2 (Additional file 1: Table S2). These SNPs had potential effects on a total of 13,838 genes (32.3% of pear genes; Additional file 1: Table S3). The proportion of non-synonymous SNPs in coding regions of pear (7.7%) and apple (10.5%) [16] was higher than that detected in either soybean [17] (1.9%) or maize [18] (0.66%), underscoring the presence of higher levels of genetic variation in pear and other fruit trees compared to some annual crops (Additional file 1: Note 2). To validate SNP calling, both PCR amplification and Sanger sequencing were conducted for 510 randomly selected SNPs in 55 pear accessions, and a 97.5% consistency for SNP calling was obtained (Additional file 3).

### Nucleotide diversity and linkage disequilibrium in genomes of Asian and European pears

The nucleotide diversity (ϴπ) of pear at the whole-genome level across all pear accessions was 5.5 × 10^− 3^ (Table 1). This was higher than those reported for other perennial crops such as peach (1.5 × 10^− 3^) [19], cassava (2.6 × 10^− 3^) [20], and grapevine (5.1 × 10^− 3^) [21], but lower than that reported for date palm (9.2 × 10^− 3^) [22]. Notably, both wild and cultivated Asian pears had higher nucleotide diversity (5.21 × 10^− 3^ and 4.76 × 10^− 3^, respectively) than either wild (3.57 × 10^− 3^) or cultivated (3.53 × 10^− 3^) European pears. However, in both Asian and European pears, similar levels of nucleotide diversities were detected for wild and cultivated accessions. This is in sharp contrast to findings reported in both soybean [17] and rice [23], wherein strong positive selection has contributed to wide differences in nucleotide diversity observed between wild and cultivated populations [24].

**Fig. 1:**
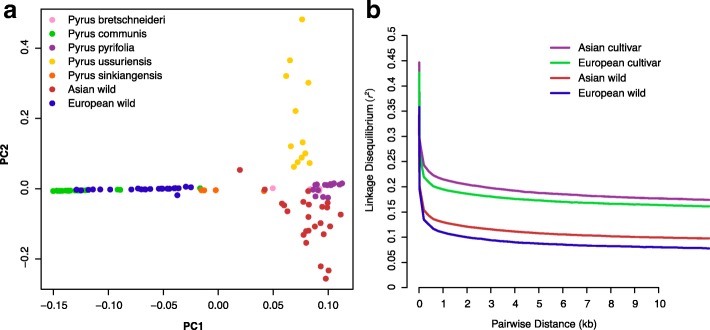
PCA and LD analysis of 113 cultivated and wild pear accessions based on whole-genome SNP analysis. **a** PCA plots of the first two eigenvectors of all 113 pear accessions. **b** LD decay determined by correlation of allele frequencies (*r*^2^) against distance (kb) among polymorphic SNP sites in different pear groups, including cultivated Asian (*red*), cultivated European (*light blue*), wild Asian (*blue*), and wild European (*green*)

To explore relationships among various cultivated and wild pear accessions, principal component analysis (PCA) of all 113 accessions was conducted using ~ 18 M SNPs, which revealed the presence of two distinct groups, namely Asian and European pears (Fig. 1a). Linkage disequilibrium (LD) analysis showed that pear genomes have relatively short LD distances and relatively rapid LD decays (Fig. 1b). The average *r*^*2*^ value among pear SNPs, corresponding to LD levels of the population, was relatively low (0.386; Additional file 1: Table S4). The average distance over which LD decayed to ~ 50% of its maximum value in pear was very short, 553 bp in cultivated Asian pears and 154 bp in cultivated European pears. *r*^*2*^ values for cultivated groups were higher for wild accessions of both Asian and European pears (Additional file 1: Table S4). Moreover, cultivated Asian pears showed the highest LD, followed by cultivated European pears. The LD decay distances for pears (211 bp) and apples (161 bp) [25] are much shorter than those reported for annual crops, such as soybean (~ 150 kb) [16] and rice (123 kb) [23].

Our findings of similar levels of nucleotide diversity between wild and cultivated pears, short LD distances, and rapid LD decay in cultivated pears all support relatively weak selection during pear domestication. This could be explained by high outcrossing rates that are maintained by self-incompatibility, as well as the short domestication history and long generation time of this perennial fruit crop [26]. Furthermore, in view of the fact that the major method for propagation of pear cultivars is by grafting, this would contribute to low numbers of sexual generations during the domestication history, and would also contribute to the weak selection during pear domestication.

### The phylogeny and structure of Asian and European pear populations

**Fig. 2:**
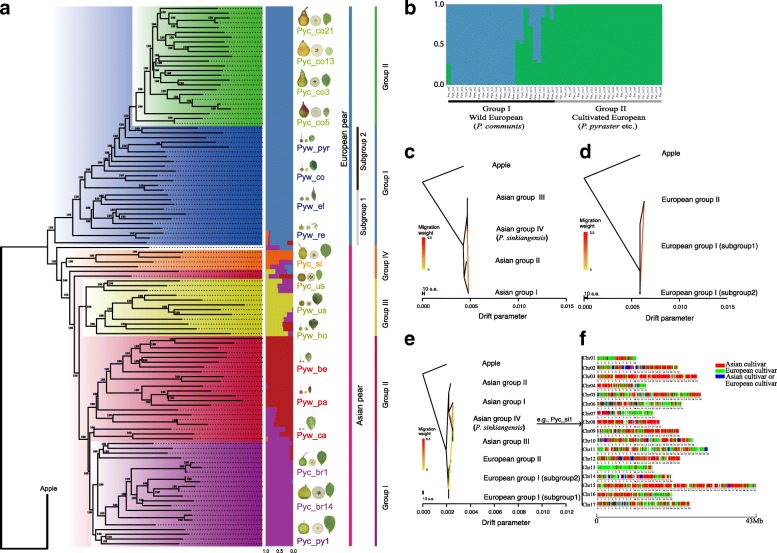
Phylogenetic tree and gene flow analysis. **a** Phylogenetic tree and the population structure (K = 5) of all 113 pear accessions inferred from whole-genome SNPs, with apple (*Malus× domestica*) used as an outgroup. Each color corresponds to a single population as noted. In population structure, each accession is represented by a *horizontal bar*. *Pyw* and *Pyc* indicate wild and cultivated accessions, respectively, and other codes correspond to abbreviated names of pear species. **b** Population structure (K = 2) of European pears. **c** Detection of gene flow within Asian pears. **d** Detection of gene flow within European pears; subgroup 1 and subgroup 2. **e** Detection of gene flow between Asian and European pears. *Lines* represent gene flow; *arrows* indicate the direction of gene flow. The *scale bar* shows a tenfold average standard error of the entries in the sample covariance matrix. The *color bar* shows the migration weight: a *red color* denotes a strong gene flow, while a *yellow color* denotes a weak gene flow. **f** IBD analysis exploring the genetic background of *P. sinkiangensis* from a combination of Asian and European pears. Blocks originating from Asian and European pears were identified in *P. sinkiangensis* Pyc-si1

A phylogenetic analysis indicated that pear accessions were clustered into two groups, Asian and European pears. This was consistent with the result of the Δ*K* analysis, which revealed an optimal K of 2 in the pear populations (Additional file 1: Figure S3), and was also similar to the pattern observed in our PCA score plot (Fig. 1a). Both phylogenetic analysis and population structure analysis (K = 5) revealed that Asian pear accessions were clustered into four groups (Fig. 2a). Asian group I consisted of the largest number of accessions and included accessions from both *P. bretschneideri* and *P. pyrifolia*. Asian group II included wild accessions from China, Japan, and Korea. Notably, the close phylogenetic relationship of cultivated *P. bretschneideri* and *P. pyrifolia* with wild *P. pyrifolia* provided whole-genome-level evidence to support the hypothesis that the two cultivated species of Asian pear, *P. bretschneideri* and *P. pyrifolia*, were derived from a common ancestor, the wild *P. pyrifolia* [27, 28]. Asian group III included both cultivated and wild accessions of *P. ussuriensis*, and these accessions were adapted to and cultivated in extremely cold regions of China. Finally, Asian group IV included all cultivated accessions derived from *P. sinkiangensis*, and revealing an admixed genetic background between Asian and European pears. Thus, it was not surprising that the Pyc-co6 accession, a recently bred hybrid from a cross of a cultivated Asian pear and a cultivated European pear, clustered in between the Asian and European groups.

On the other hand, the phylogenetic analysis revealed that European cultivated pears formed a clade that was nested within wild European pear accessions (Fig. 2a). However, it was interesting to note that there was little change in the population structure for European pears, with increasing K values from 2 to 7 (Additional file 1: Figure S3a). In view of the high polymorphism and diversity of Asian pears, which might influence the population structure of European pears, the population structure analysis for European pears was conducted independently. It was found that European pears could be classified into two groups (Fig. 2b). European group I included wild accessions from Europe and North Africa. Almost all of the cultivated European pears clustered together into European group II, except for the wild accession Pyw-ni1 belonging to *P. nivalis*, an atypical pear used in the production of “perry” cider. Among 13 wild accessions in European group I, *P. pyraster* accessions were those most closely related to European group II accessions. Thus, it seems that *P. pyraster*, which grows widely throughout Europe [29], is likely the progenitor species from which cultivated European pears are derived. It is important to note that very few changes in clustering of European pears were observed at different K values (Additional file 1: Figure S3a). This is supported by findings obtained from ϴπ analyses revealing that, relative to sampled Asian pears, there was lower genetic diversity present among sampled European pear accessions. Thus, for subsequent gene flow and identity-by-descent (IBD) analyses, European group I accessions were split into two subgroups based on their geographical sampling locations. European group I subgroup 1 included accessions that were relatively closer to east Asia, while European group I subgroup 2 accessions were relatively farther away from east Asia (Additional file 1: Table S5; Fig. 2a).

Subsequently, gene flow within and between Asian and European pears were explored. First, we used groups from the phylogenetic tree to conduct a gene flow analysis using Treemix within Asian pears. While we detected a relatively strong gene flow from Asian group I to Asian group IV (*P. sinkiangensis*), we did not detect significant gene flow among the other pairings of Asian pear groups (Fig. 2c). As for European pears, extensive gene flow (*P* value = 2.2e-308; F_statistic = 0.988) was detected between European group II and European group I subgroup 2 (Fig. 2d). To further investigate gene flow between Asian and European pears, an analysis was conducted using all groups with no significant gene flow between Asian and European groups. A weak gene flow was detected from European pear group accessions to the Asian pear accession of *P. sinkiangensis* (Fig. 2e). All these findings are consistent with the earlier hypothesis that *P. sinkiangensis* is derived from a hybridization between Asian and European pears.

An IBD analysis of Asian group IV (*P. sinkiangensis*) was conducted to verify gene flow into this species from both cultivated Asian and cultivated European pears (Fig. 2f). It was observed that the proportion of genetic background from Asian cultivated pears was 45.3–61.8% in *P. sinkiangensis*, which was higher than that detected from European group II (17.9–35.3%). IBD analysis of European group I revealed that *P. sinkiangensis* contained 10.9–23.0 and 11.8–26.7% of the genetic backgrounds of European subgroups 1 and 2, respectively. This was lower than that detected for European group II, thus indicating that cultivated European pears contributed a higher proportion of genetic background to *P. sinkiangensis* compared with wild European pears. Therefore, this IBD analysis further supported Treemix results noting that *P. sinkiangensis* was the product of a hybridization that occurred between cultivated Asian and cultivated European pears. These findings are reasonable to expect from a historical perspective, as there was extensive cultural contact along the Silk Road from 207 BCE to 220 CE [30]. Interestingly, there is a historical record from about 2000 years ago of a Han dynasty diplomat, Qian Zhang, bringing over cultivated Asian pear to the Xinjiang region [31]. Given our finding that the admixed *P. sinkiangensis* species must have resulted from hybridization between cultivated Asian and cultivated European pears, we can speculate that historical and commercial influences may have contributed to the development of this unique species of cultivated pear.

### Origin and dissemination of wild pears

**Fig. 3:**
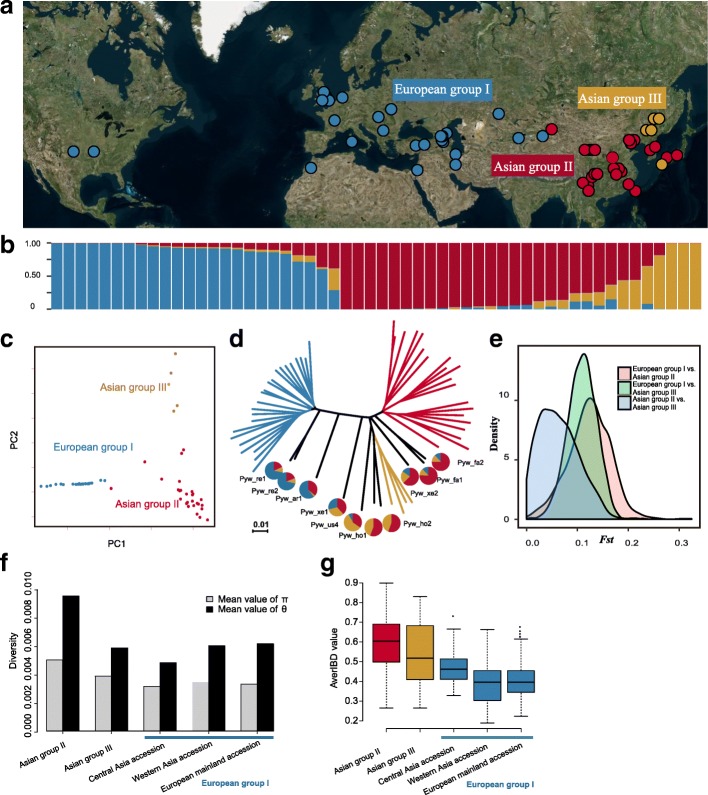
Genetic relationships of wild pears in different geographical regions. **a** Wild pear distribution in different geographical regions. **b** Population structure (K = 3) of all 57 wild accessions. Each color corresponds to a single population as noted. Each accession is represented by a *vertical bar*. Different color represents the probability of an accession belonging to a different genetic background. **c** PCA plots of wild accessions. **d** Phylogenetic tree of wild pear accessions and admixed genetic component of some species. **e** Distribution of *F*_ST_ values between three major wild groups. **f** ϴπ values of different pear groups. Asian group II, wild accessions distributed in south and west regions of China; Asian group III, wild accessions distributed in the northeast region of China. European group I was split into three subgroups: Central Asia, West Asia, and the European mainland. **g** Lineage homologies of wild accessions of both Asian and European pears by identity-by-descent (IBD)

To explore the origin and dissemination of pears, we divided the 57 wild pear accessions into three groups, which were the same as the aforementioned European group I, Asian group II, and Asian group III groups (Fig. 3a). The filtered SNP dataset of 57 wild pear accessions (excluding all cultivated pear accessions to avoid gene flow effects), consisting of 16,320,215 SNPs, were subjected to both population structure analysis (Fig. 3b) and PCA (Fig. 3c). Results strongly supported a population structure of K = 3. Of particular note, *P. ussuriensis* accessions that comprised Asian group III, known to be highly cold-tolerant, were collected from extremely cold environments in what is currently the northeast region of China. Mantel tests showed that this population structure was highly correlated with geographical distribution (*P* = 1e-04). This suggests that geographical factors have been highly influential in generating the observed genetic variation present among wild pear populations across the Eurasian continent. Interestingly, this also strongly suggests that several accessions originally annotated to belong to *P. regelii*, *P. armeniacaefolia*, *P. xerophila*, *P. hopeiensis*, and *P. fauriei* species were instead highly admixed (Fig. 3d). This finding was not surprising as these “species” were not reproductively isolated. Therefore, it is likely that inter-“species” hybridizations and genetic admixtures occurred among these pears.

Levels of population differentiation, *F*_ST_, were then estimated across all chromosomes among the three groups of wild accessions (Fig. 3e). The *F*_ST_ between Asian group III and Asian group II was smaller than that between European group I and Asian group II. This suggested that the divergence of Asian and European groups preceded the divergence of Asian group II from Asian group III. Furthermore, when ϴπ analysis was conducted to evaluate levels of genetic diversity of these groups, it was found that Asian group II and Asian group III showed the highest levels of diversity (Fig. 3f) when compared to other groups.

In addition, IBD analysis was conducted to assess the same DNA segments within and across accessions (Fig. 3g). Overall, IBD values were higher for accessions in both Asian group II (0.59) and Asian group III (0.54), followed by those found in European group I, including central Asia (0.47), Western Asia (0.38), and the European mainland (0.439). Results of population structure analyses of wild pear accessions along with their geographical distributions support the hypothesis that pear must have originated in what is now known as the southwest region of China. Subsequently, it was then disseminated throughout central Asia before it was further spread over to western Asia and then to Europe.

### A proposed model for the evolutionary pathway of pear

**Fig. 4:**
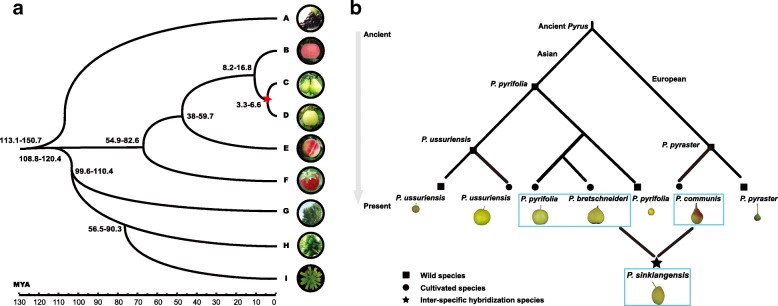
Genetic relationships and divergence times of pear species. **a** Genetic relationships of wild and cultivated pear species. **b** Divergence time of Asian and European pears. *A*, *Vitis vinifera*; *B*, *Malus* × *domestica*; *C*, *Pyrus communis*; *D*, *Pyrus bretschneideri*; *E*, *Prunus persica*; *F*, *Fragaria vesca*; *G*, *Populus trichocarpa*; *H*, *Carica papaya*; and *I*, *Arabidopsis thaliana*

The divergence time of Asian and European pears was estimated by constructing a phylogenetic tree using a total of 420 single-copy conserved genes from nine plant species (Fig. 4a). It was estimated that Asian and European pears diverged between 6.6 and 3.3 MYA, far prior to any possible human intervention, perhaps mediated by animals through the dispersal of fruit and pollen. Based on the phylogeny, population structure, gene flow, and IBD analyses, we propose the following model for divergence and independent domestication of Asian and European pears (Fig. 4b).

The hypothetical common ancestor of both Asian and European pears seems to have originated in China, subsequently disseminated through central Asia, and then eventually on to western Asia and Europe. Considering the fact that there is no reproductive isolation in the population, and given that pear is a typical self-incompatible species and an obligate outcrosser, it is likely that a “continent-wide species” must have undergone local adaptation followed by independent domestication processes for each of Asian and European pears. Each of these domestication processes must have involved selection for distinct phenotypic traits, including distinctive fruit shape, flavor, and texture traits that are now characteristic of Asian and European pears [32, 33].

This proposed model clarifies present-day complex relationships among the large numbers of so-called pear species. The five currently recognized cultivated pear species have been domesticated from three wild species. This is quite different from other crops which are often domesticated from a single wild species [34, 35]. The only species of European pear, *P. communis*, is derived from a wild European species, *P. pyraster*. One of the four species of Asian pear, the cultivated *P. ussuriensis*, is derived from the wild *P. ussuriensis*. Two other cultivated species of Asian pear, *P. pyrifolia* and *P. bretschneideri*, are derived from a common ancestor, the wild *P. pyrifolia*. Finally, the admixed species of the fourth cultivated Asian pear, *P. sinkiangensis*, is derived from hybridization that must have occurred within the last 3000 years between the cultivated European pear (*P. communis*) and the cultivated Asian pear, either *P. pyrifolia* or *P. bretschneideri*. The IBD analysis indicates similarly sized genome contributions from *P. pyrifolia* and *P. bretschneideri* to *P. sinkiangensis*.

### Balancing selection along with rapid evolution of *S-RNase* genes have strengthened self-incompatibility in pear

Pear exhibits typical gametophytic self-incompatibility (GSI), which is controlled by a single multi-allelic locus, the *S*-locus. The *S*-locus contains the pistil determinant, *S-RNase*, and candidate pollen determinant *S*-locus haplotype F-box genes, *SFB* genes [14, 36]. It is commonly known that the *S-RNase* gene exhibits high sequence variability among different pear cultivars [37, 38].

To analyze allelic diversity of the *S-RNase* gene, cleaned reads of each pear accession were mapped onto the *S*-*RNase* locus of the reference pear genome of ‘Dangshansuli’. A total of 92 SNPs were detected among *S-RNase* alleles of wild accessions and 141 SNPs among *S-RNase* alleles of cultivated accessions of pear. Mean ϴπ values were 1.70 × 10^− 1^ for wild accessions and 1.72 × 10^− 1^ for cultivated accessions (Additional file 1: Table S6). These mean ϴπ values were much higher than the mean diversity of the genes (1.56 × 10^− 2^) detected in the whole genome. Notably, the high genetic diversity of *S-RNase* alleles was almost identical in both cultivated and wild accessions (Additional file 4), suggesting that this gene has not experienced strong selection pressure under human intervention. Meanwhile, both cultivated and wild Asian and European pears had positive *Tajima’s D* values (> 2.0) for the *S-RNase* gene. This indicated that a balancing selection must have contributed to maintenance of high levels of polymorphisms. This finding was also supported by high π and *F*_ST_ values obtained for both Asian and European pears (Additional file 1: Table S7).

We speculated that a fast evolution might help to account for the wide variability observed in the *S-RNase* gene. Therefore, the evolution rates of *S-RNase* and other genes under balancing selection were compared. The evolution rate of the *S-RNase* gene of pear was estimated to be at least 1.91e^− 09^ sites/year, whereas those for other genes under balancing selection ranged between 2.31e^− 10^ and 6.10e^− 10^ sites/year. Therefore, the evolution rate of the *S-RNase* gene remains higher than the estimated evolution rates of other balance-selected genes (Additional file 1: Note 3). These findings support the hypothesis that rapid evolution of the *S-RNase* gene may have led to its high variability, which is consistent with the theory that reproduction-related genes show higher evolution rates [39, 40]. This has likely contributed to strengthening of GSI and promoting outcrossing, thus facilitating genetic recombination among genotypes of different genetic backgrounds of pear.

### Independent domestication processes for each of Asian and European pears

**Fig. 5:**
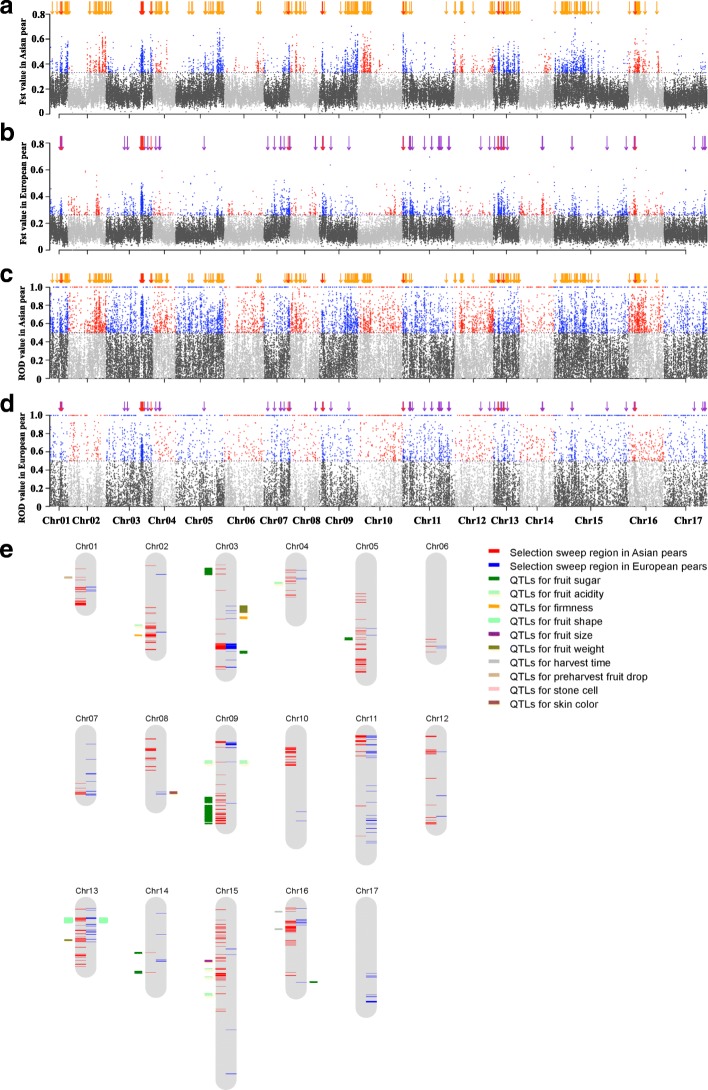
Distinct domestication signals in Asian and European pears. **a** Distribution of *F*_ST_ values across the whole genome of Asian pears. **b** Distribution of *F*_ST_ values across the whole genome of European pears. **c** Distribution of ROD values across the whole genome of Asian pears. **d** Distribution of ROD values across the whole genome of European pears. *Yellow arrows* indicate genes in selective sweeps of only Asian pears. *Purple arrows* indicate genes in selective sweeps of only European pears. *Red arrows* indicate genes in selective sweeps common to both the Asian and European pears. The *horizontal dotted line* indicates the threshold of *F*_ST_ 5% and ROD > 0.5, respectively. **e** Overlap of selective sweeps and QTLs related to fruit traits in pear. The *inside lines* of each linkage group indicate selective sweeps, while the *outside lines* of each linkage group indicate QTLs. A total of 208 selective sweeps in Asian pears showed coincidence with QTLs related to fruit traits, including sugar, acidity, stone cell, firmness, fruit size, fruit shape, as well as traits for preharvest fruit drop and fruit harvest time. A total of 14 selective sweeps in European pears showed coincidence with QTLs related to fruit traits, including sugar, acidity, firmness, fruit size, fruit shape, and skin color

Human intervention via artificial selection of favorable phenotypic traits to enhance production and improve desirable agronomic traits can both reduce levels of genetic variability and skew allele frequencies [41]. Separate selective sweeps driven by artificial selection were detected in Asian and European pears. For Asian pears, selective sweep signatures for a total of 9.29 Mb of the genome sequence, containing 857 putative genes, were detected. For European pears, there were selective sweep signatures for 5.35 Mb of the genome sequence containing 248 putative genes (Fig. 5a-d; Additional file 5). It was notable that there was only 515 kb of overlap for regions with selective sweep signatures between Asian and European pear genomes, containing 47 putative genes (Additional file 6).

The different genes identified in selective sweeps of Asian and European pears were found to be enriched for 47 and 34 biological processes, respectively, including growth, response to cold, meristem and flower development, and single-organism metabolic processes, which could be involved in the distinct domestication pathways that have contributed to different traits of Asian and European pears. For example, in Asian pears, 11 cell wall degradation-related genes were found in selective sweep regions (Additional file 7), while none were found in selective sweep regions of European pears. These domestication-related genes might contribute to the crisp fruit flesh texture observed in Asian pears, compared with the soft and fine flesh texture of European pears. Four genes associated with fruit size, including one YABBY (*Pbr003157.1*; Additional file 1: Figure S4), two cyclin-like genes (*Pbr015160.2* and *Pbr028956.1*), and one EXP4 (expansin-A4-like, *Pbr041772.1*), were found in selective sweeps of Asian pears. In contrast, two different fruit size-related genes, *Pbr012098.1* and *Pbr012099.1*, which are homologous to tomato *fw2.2* [42], were found in selective sweeps of European pears. This result indicates that different genome regions were selected for fruit size in Asian and European pears.

**Fig. 6:**
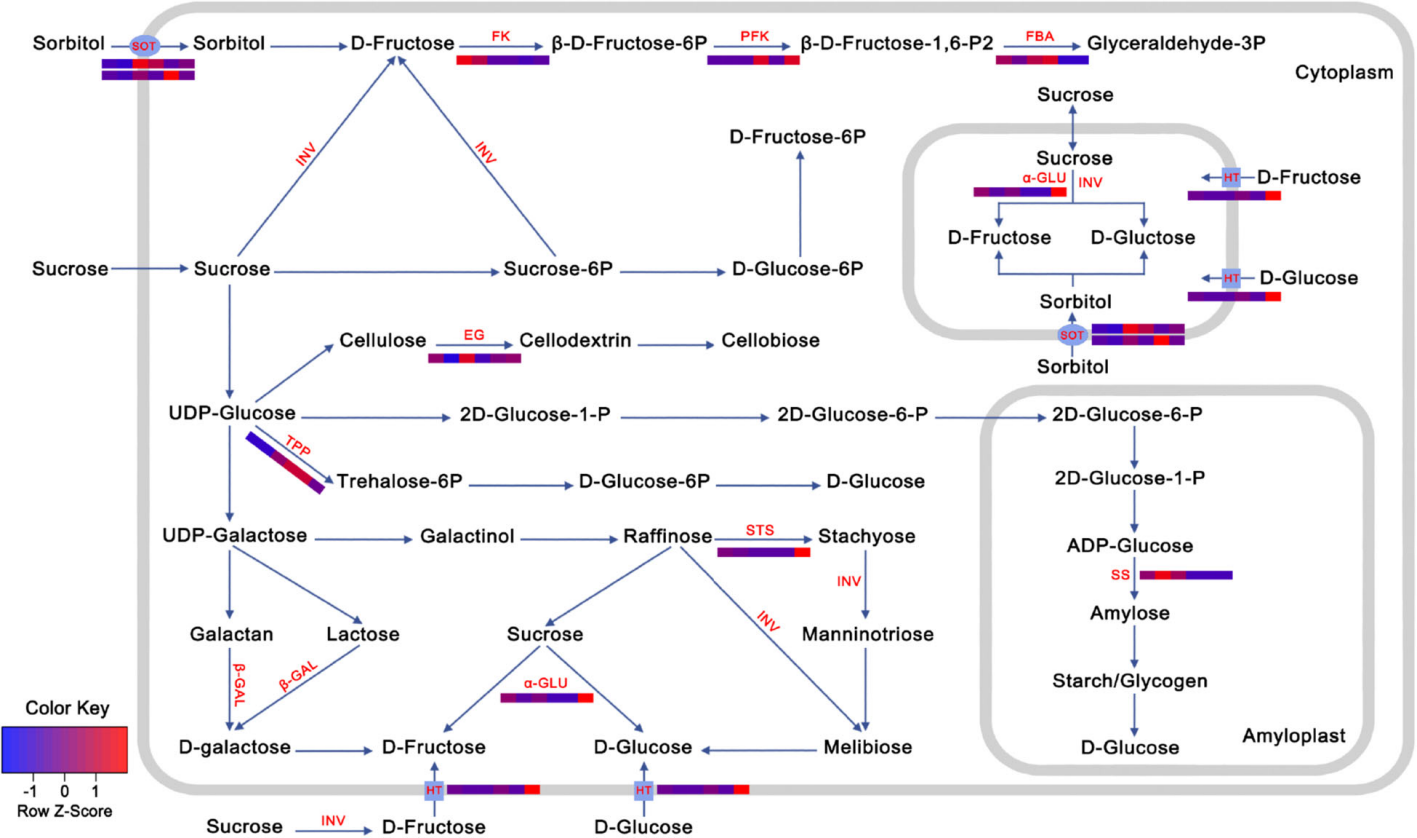
Sugar metabolism-related genes associated with domestication of pear. Genes in *red* correspond to genes in selective sweep regions. Transcriptome data are derived from wild and cultivated pear fruits. The two wild pears are ‘Baitanggengzi’ and ‘Tiantanggengzi’ (from *left* to *right*), while the four cultivated pears are ‘Yali’, ‘Hosui’, ‘Nanguo’, and ‘Starkrimson’ (from *left* to *right*). *FK* fructokinase, *PFK* phosphofructokinase, *FBA* fructose-bisphosphate aldolase, *INV* beta-fructofuranosidase, *EG* endoglucanase, *TPP* trehalose-phosphatase, *STS* stachyose synthetase, *β-GAL* beta-galactosidase, *α-GLU* alpha-glucosidase, *SS* starch synthase, *SOT* sorbitol transporter, *HT* hexose transporter

Many sugar-related genes were found in selective sweeps, indicating a preference for sweet fruit during domestication. For Asian pears, a total of 45 sugar-related genes were identified in the selected regions, including four genes (*Pbr000142.1*, *Pbr019272.1*, *Pbr018801.2*, and *Pbr030762.1*) that encode enzymes (starch synthase, fructokinase, and invertase) involved in sugar metabolism, and three genes (*Pbr013451.1*, *Pbr037348.1*, and *Pbr037349.1*) that encode sorbitol and hexose transporters (Additional file 1: Figure S5). Comparatively, only 11 sugar-related genes were identified in the selected sweeps of European pears, including *Pbr039977.1*, encoding a sorbitol transporter. Of these genes, *sorbitol transporter* (*SOT*; *Pbr013451.1*), *starch synthase* (*SS*; *Pbr000142.1*), *trehalose-phosphatase* (*TPP*; *Pbr000530.1*), and *endoglucanase* (*EG*; *Pbr030853.1*) were expressed at higher levels in the ripe fruits of cultivars than in those of wild accessions (Fig. 6), indicating that these genes could be important candidates associated with sugar transport and biosynthesis in pear fruits. In contrast, *fructokinase* (*FK*; *Pbr018801.2*) displayed lower expression in the ripe fruits of cultivars than in those of wile accessions, leading to a reduction of D-fructose transformed to β-D-fructose-6P, and thus promoting sugar accumulation in the fruits of cultivars.

Fewer genes involved in organic acid metabolism were identified in selective sweeps. The citric acid-related gene *Pbr014969.1* (Additional file 1: Figure S6), a homolog of *ACLA-3* that controls the synthesis of citrate [43], was identified in Asian pears, whereas three malate acid biosynthesis or transport-related genes, *Pbr013232.1*, *Pbr013272.1*, and *Pbr030186.1*, were identified in European pears. These findings further support an earlier observation regarding the presence of different dominant acid components in Asian and European pears [44].

Stone cells specifically accumulate in pear flesh and can detract from eating quality. The stone cell content shows a close relationship with biosynthesis, transfer, and deposition of lignin in cell walls. Six genes related to lignin biosynthesis in the selective sweep regions (Additional file 1: Figure S7), including two peroxidases (*POD*; *Pbr000146.1* and *Pbr15965.1*), two hydroxycinnamoyl transferases (*HCT*; *Pbr006408.1* and *Pbr012356.2*), one ferulate-5-hydroxylase (*F5H*; *Pbr031416.1*), and one cinnamoyl-CoA reductase (*CCR*; *Pbr039962.1*), were found in Asian pears, while only two *CCR* genes (*Pbr013290.1* and *Pbr039962.1*) were detected in European pears. This finding may help to explain the higher concentration of stone cells in Asian pears than European pears.

As for volatile compounds in pear fruit, a total of 12 and three genes annotated in fatty acid metabolism pathways were identified in selective sweeps of Asian and European pears, respectively. Four alcohol dehydrogenase genes (*Pbr003230.1*, *Pbr027590.1*, *Pbr027591.1*, and *Pbr034873.1*) related to aroma biosynthesis were detected in Asian pears, while only two (*Pbr013212.1* and *Pbr028181.1*) were found in European pears, indicating that aroma in these two groups of pears is regulated by different genes in the metabolic pathway.

It is interesting to note that the 47 genes in selective sweeps of both Asian and European pears include one *POD* gene (*Pbr013214.1*) related to stone cell formation and one cyclin-like gene (*Pbr035650.1*) related to fruit size development, suggesting a certain degree of convergent domestication of fruit quality in Asian and European pears.

Since the selective sweep signatures could colocalize with many important agronomic traits, potentially some of which have been implicated in quantitative trait loci (QTL) studies. We looked for enrichment in selective sweep signatures and candidate QTL regions. The selected regions included previously reported QTL with accurate chromosome information from hybridized segregating populations of pear [45–47] and a newly constructed pear population (“Niikata” × “Hongxiangsu”; Additional file 8). We found 208 and 14 selective sweeps from Asian and European pears that overlapped, respectively, with QTL regions (permutation test *P* values of 1E-5 and 0.35, respectively), indicating significant colocalization signals in Asian pears. The weak colocalization signals in European pears might be due to the fact that the QTL used here were mostly identified from Asian pear, while few fruit related QTL have been reported in European pears (Fig. 5e). In the overlapping regions of QTL and selective sweeps, a total of 151 and 17 genes were identified in selective sweeps of Asian pears and European pears, respectively (Additional file 9). Among them, 94 genes were mapped to sugar-related QTL, 20 to fruit size, 14 to acidity, ten to firmness, 18 to fruit shape, and three to stone cell content. These results strongly support that genes with selective sweep signatures in QTL regions might play important roles to regulate the distinct phenotypic traits selected in Asian versus European pears.

## Conclusions

In this study, we report on genome variation mapping of 113 wild and cultivated pear accessions, collected from worldwide germplasm material. Our findings provide insights that informed our proposed model for the divergence, dissemination, and independent domestication of Asian and European pears. A rapid LD decay was identified in pear, thus revealing a characteristic weak domestication process for this perennial fruit tree. Separate selective sweep signatures identified between Asian and European pears underlined the distinct phenotypic traits observed in pear, including fruit acidity, sugar, and stone cell content, among others. Population structure analysis provided new evidence to support the admixed genetic background of some pear species, which was likely driven by self-incompatibility. Furthermore, analysis of the nucleotide diversity of the *S-RNase* gene controlling self-incompatibility suggested that a potential mechanism which promoted outcrossing must have accounted for the extensive genome diversity observed in pear.

Finally, it bears repeating that this study offers an unprecedentedly large amount of genomic resources for wild and cultivated pears. This, alongside our identification of candidate genes in selective sweep regions and colocalized QTLs, will significantly contribute to efforts for genetic improvement and molecular breeding of pear. Further, these findings raise intriguing questions that will almost certainly set the stage for the next phase of global pear and perennial tree fruit research.

## Methods

### Sampling information and sequencing

In this study, a total of 113 accessions, belonging to 33 *Pyrus* species from 26 countries and spanning a wide geographic distribution, were collected and sequenced. This collection covers accessions from all five of the major cultivated species of pear and from most recognized wild species (Additional file 2 and Additional file 1: Figure S1).

Genomic DNA was extracted from leaves using the CTAB method. Paired-end DNA libraries with short inserts (~ 500 bp) were constructed according to the manufacturer’s instructions and sequenced using the HiSeq™ 2000 or Hiseq™ 4000 platforms (Illumina, USA). To retain reads of high quality, reads with fewer than 5% N (missing) bases and with fewer than 50% of bases of base quality < 5 were deemed as cleaned reads. All other reads were discarded.

### Reference genome selection and SNP calling

First, to facilitate the selection of an appropriate reference genome, we performed comparison of two published pear genomes: the Asian pear genome ‘Dangshansuli’ and the European pear genome Bartlett [14, 15]. For the details of our assembly quality assessment based on the contig N50, the scaffold N50, and the scaffold size values, as well scaffold to chromosome anchoring ratios, see Additional file 1: Table S1. Second, to clarify differences between these two potential reference genomes, we conducted synteny analysis of both genomes and also used all-versus-all BLASTP (E-value less than 1e-5) analysis to identify orthologous genes of the two genomes. Here, an orthologous gene was defined as a positive reciprocal BLASTP hit between the two genomes. MCScanX [48] was used to analyze synteny blocks. Third, for SNP calling, we used ‘Dangshansuli’ as the reference genome and the following protocol: 1) SOAPaligner (version 2.22 beta) [49] was used to map cleaned reads to the pear reference genome [14]; 2) based on genome coordinates and following removal of potential PCR duplicates, alignments were used to build a consensus sequence for each accession using SOAPsnp (version 1.04) [50]; 3) further filtration was conducted to obtain an accurate genotype for each site in each accession using the following criteria: (a) the quality value should be more than 20, (b) the number of unique reads for a confirmed genotype should be higher than 2, and (c) the copy number for each site had to be less than 1.5; and 4) the confirmed credible genotype from all accessions for each site and biallelic SNPs with missing rates of less than 0.5 were deemed as SNP variants in the population. Further, to ensure that the variant mapping rates of divergent samples would not deleteriously affect the analysis of θπ, θw, and *Tajima’s D* and so on, SNPs that were present in the syntenic blocks and that had a missing rate of < 10% among the accessions for both Asian pears and European pears were selected to validate the findings, yielding results that were consistent with mapping to the ‘Dangshansuli’ Asian pear genome (Additional file 1: Note 1). This four-step process led us to ultimately select ‘Dangshansuli’ as the reference genome. A total of 510 SNP loci were randomly selected, and Primer 3 was used to design primers for PCR-based sequence verification. Following PCR amplification, fragments were Sanger sequenced by Invitrogen Inc. (USA).

### Population genetics analysis

Genetic distances determined in analyses of 113 accessions and 57 wild accessions were calculated by sampling with replacement SNPs (200 times) using the p-distance method [23], and neighbor-joining trees were constructed using the neighbor program in the EMBOSS toolbox [51]. Trees were then merged, and Figtree (http://tree.bio.ed.ac.uk/software/figtree/) was used to adjust the neighbor-joining tree. A principal component analysis (PCA) was conducted using the eigen function in R base to obtain an eigenvector. The top four eigenvectors of samples were plotted using the ggplot2 R package [52]. FRAPPE [53] was used to infer the population structure among samples, wherein the maximum iteration time was set to 10,000, and the number of population groups (K) was varied from 2 to 5. To determine the most appropriate population structure’s classification for all 113 accessions, FRAPPE analysis [53] was performed 20 times on 1000 randomly selected SNPs at 4dTv (four-fold degenerate site) for each K value from 1 to 10 according to Evanno G et al. [54].

To estimate the gene flow between Asian and European pears, both wild and cultivated pears, 4.7 M SNPs were selected with the following criteria: the missing rate was < 0.9 in both Asian and European pears. Based on these SNPs, we used Treemix version 1.13 to investigate the gene flow between groups/subgroups, with the settings: “-se -bootstrap -k 500 -m”, wherein the number (−m) varied from 1 to 5.

### Diversity analysis

Nucleotide diversity analyses were conducted, including the average pairwise divergence within a population (θπ) [41], the Watterson’s estimator (θw) [55], and *Tajima’s D* [41]. A sliding window of 10 kb, along with a step of 5 kb, was used to estimate the θπ, θw, and *Tajima’s D* values. For each window, these values were calculated using an in-house Perl script with the Bio::PopGen package. Pairwise *F*_ST_ values [56] were computed in the same windows to measure the population differentiation between groups. We also calculated the nucleotide diversity for various types of genomic regions (mRNA, CDS, introns, UTRs, and intergenic regions).

### LD and LD blocks

Correlation coefficients (*r*^*2*^) of alleles were calculated using Haploview [57] to measure LD values in each of the four pear populations (i.e., Asian cultivated, Asian wild, European cultivated, and European wild). The parameters were set as follows: -maxdistance 200, -dprime, -minGeno 0.6, -minMAF 0.05, and -hwcutoff 0.01. LD decays were then plotted using a custom R script for each of the four pear populations. The parameter “-blockoutput GAB” was added to the Haploview program to detect LD blocks for each of the four pear populations.

### Identification of identical-by-descent segments between Asian and European pear

Using pairwise accessions, identical-by-descent (IBD) regions were identified in contiguous 10-kb windows with no overlaps. The number of SNPs in each window should be more than 10. Similarity scores were calculated using the p-distance method [23] in each of the windows. Windows with percent similarity scores higher than 95% were deemed as IBD windows. The percentage of IBD windows along the entire genome was calculated for every pair of accessions.

Based on the geographical origins of accessions used in this study, wild accessions were separated into the following five wild geographic groups: Asian group II accessions, Asian group III accessions, Central Asian accessions, Western Asian accessions, and European mainland accessions. The average percentage of IBD values in the sliding windows (AverIBD) were calculated for each of the five wild geographic groups using the following formula:

$$ \mathrm{AverIBD}=\frac{1}{k}\sum \limits_{i=1}^n\sum \limits_{j=1}^m{P}_{ij} $$

where *n* corresponds to the number of accessions for a geographical group, *m* corresponds to the number of all Asian wild pear accessions, *P*_*ij*_ is the percentage of IBDs in the genome for a pair of accessions (each from a different groups), and *k* is the count of *P*_*ij*_ between a geographical group and all Asian wild pear accessions.

### Self-incompatibility gene analysis

By mapping all pear sequencing reads to identified *S-RNase* alleles using *bwa* version 0.7.12-r1039) [58], cleaned reads specific for *S-RNase* genes were identified and used to call SNPs using the GATK package Haplotype Caller [59]. Based on these called SNPs, θw and θπ, which indicate the nucleotide diversity, of *S-RNase* genes were calculated for different pear groups. The evolution rate of *S-RNase* and other genes under balancing selection were calculated using the d/2 T formula, where d is the nucleotide diversity and T is the duration of the time since divergence from the most recent common ancestor.

### Divergence time of Asian and European pears

Various plant species, including *Vitis vinifera* (common European grape) [60], *Carica papaya* (papaya) [61], *Fragaria vesca* (woodland strawberry) [62], *Prunus persica* (peach) [63], *Malus* x *domestica* (cultivated apple) [64], *Arabidopsis thaliana* [65], and *Populus trichocarpa* (black cottonwood) [66], were used to estimate the divergence time of cultivated Chinese pears (*P. bretschneideri*) [14] from cultivated European pears (*Pyrus communis*) [15]*.* A total of 420 single-copy gene families in all nine species were identified. Based on 4dTv (four-fold degenerate sites) in these 420 single-copy gene families, a phylogenetic tree was constructed using PhyML (v3.0) [67]. Based on this phylogenetic tree and known divergence time range between *Populus trichocarpa* and *Arabidopsis thaliana* (100–120 MYA), we used MCMCTREE (PAML version 4.l4) to estimate the divergence time between cultivated Asian and cultivated European pears [68, 69].

### Selective signals in pear

First, we removed the admixed genotypes (Additional file 1: Table S8) based on population structure analysis (Fig. 2a). Then, for Asian pears, cultivated accessions of *P. pyrifolia* and *P. bretschneideri* were used to detect selective sweeps. This strategy was used due to the admixture nature of *P. sinkiangensis* and availability of a limited number of *P. ussuriensis* accessions (Additional file 1: Table S8). Finally, 89 pear accessions including 19 wild Asian, 22 cultivated Asian, 24 wild European, and 24 cultivated European accessions (Additional file 10) were used for selective sweep analysis.

SNPs with missing rates of less than 0.5 in both Asian and European pears were deemed as common SNPs for selection sweep analysis. To identify regions with signals for selective sweeps in cultivated pears, θπ, *Tajima’s D*, reduction of diversity (ROD = 1 − θπ_cul_/θπ_wild_), and *F*_ST_ parameters were calculated in non-overlapping windows of 10 kb along the entire pear genome, based on common SNPs. Regions (10-kb window) with signals for selective sweeps were identified using the following criteria: among the top 5% of *F*_ST_, ROD > 0.5, and bottom 10% of *Tajima’s D* distribution. Regions with balancing selection were identified using the bottom 5% of *F*_ST_, and top 5% of *Tajima’s D*, and the top 10% of θπ.

### RNA sequencing and sequence mapping

RNA was extracted from fruit flesh for a total of 24 samples (eight species × three stages). RNA sequencing libraries were constructed using the Illumina standard mRNA-Seq Prep Kit (TruSeq RNA and DNA Sample Preparation Kits version 2). Single end RNA-Seq data were generated with length of 49 bp. Reads were filtered and trimmed and then mapped onto ‘Dangshansuli’ (*Pyrus bretschneideri*) coding sequences using SOAPaligner [49].

### Sugar content measurements

High performance liquid chromatography (HPLC) was used to measure pear fruit sugars, including sucrose, glucose, fructose, and sorbitol. Sugars were extracted from pear flesh by grinding, and then were dissolved and filtered through a SEP-C18 cartridge (Waters, WAT021515) and Sep-Pak filter. Sugars were processed using a Waters 1525 system (Waters, Shanghai, China); the column was 6.5 mm × 300 mm, inner diameter, 10 μm particle size (Waters), with a Sugar-pak 1 Guard-Pak Holder and Insert (Waters) cartridge for the guard column. Column temperature was set to 85 °C, and 35 °C was the reference cell temperature.

### Association of selective sweep regions with QTLs

Selective sweep regions were associated with QTLs identified in pears, including previously published QTLs and new QTLs related to fruit quality that we identified in the course of the present study. Two F1 populations were used for these new QTL mapping analyses, including an F1 population containing 102 individuals derived from crossing ‘Bayuehong’ × ‘Dangshansuli’ (phenotyping of different fruit traits, including sugar content, acid content, stone cell content, and fruit size, were conducted in 2014 and 2015) and an F1 pear population of 176 individuals from a cross between ‘Niikata’ × ‘Hongxiangsu’ using an 8× re-sequencing strategy (phenotyping of fruit-related traits such as sugar content, acidity content, stone cell content), which were investigated in 2015 and 2016.

MapQTL6.0 (https://www.kyazma.nl/index.php/MapQTL/) was used for linkage map construction using a regression mapping algorithm and the Kosambi function. MapQTL6.0 was also used to perform interval mapping and to conduct MQM and Kruskal-Wallis tests to evaluate candidate QTLs. Finally, markers with *p* < 0.005 and interval mapping LOD values higher than 3.5 were identified as QTLs. In comparison with the overlap of selective sweep regions and QTLs, we used enrichment tests with a sliding window size of 10 kb with 100,000 repetitions throughout the genome to find the overlap regions with candidate QTL regions.

## Additional files

Additional file 1: Supplementary data and analysis. (DOC 12492 kb)

Additional file 2: Summary of sequencing and mapping of 113 pear accessions. (XLSX 51 kb)

Additional file 3: SNPs and genotype verification by Sanger sequencing. (XLSX 76 kb)

Additional file 4: S-genotyping for different accessions. (XLSX 20 kb)

Additional file 5: List of genes in selective sweeps in Asian and European groups during domestication from wild to cultivated pears. (XLSX 188 kb)

Additional file 6: List of 47 genes in common selective sweeps of both Asian and European groups during domestication from wild to cultivated pears. (XLSX 39 kb)

Additional file 7: List of genes in selective sweep regions involved in important traits during domestication from wild to cultivated pears in Asian group. (XLSX 60 kb)

Additional file 8: List of QTL regions and association SNPs. (XLSX 48 kb)

Additional file 9: List of domesticated genes within QTL regions and association SNPs. (XLSX 62 kb)

Additional file 10: List of genes in selective sweep regions of *P. ussuriensis* during domestication from wild to cultivated pears. (XLSX 44 kb)
