# Supplementary material for: Diversification and independent domestication of Asian and European pears
Source: Genome Biol. 2018 Jun 11;19:77. doi: 10.1186/s13059-018-1452-y (PMC5996476; doi:10.1186/s13059-018-1452-y)
Supplement: Supplementary file 1 — Supplementary data and analysis. (DOC 12492 kb) [file 13059_2018_1452_MOESM1_ESM.doc]

**NOTE 1**

**Mapping rates revealed divergence of sequenced accessions with the reference genome**

The observed differences in mapping rates have indicated presence of levels of divergence between sequenced accessions and the reference genome. Sequences from cultivated (average of 72%) and wild (average of 68%) Asian accessions mapped to the reference genome at a higher rate than those of cultivated (average of 57%) and wild (average of 44%) European accessions. This was to be expected as the reference genome used in this study, ‘Dangshansuli’ (*P. bretschneideri*), is a cultivated Asian species.

In this study, considerable care was taken in the selection of ‘Dangshansuli’ over the European pear genome ‘Bartlett’ as the reference genome. This was a necessary step as there are currently two published pear genomes to choose from. A pesudo alignment between these two genomes led us to anticipate a low mapping rate for cross-species alignment. First, ‘Dangshansuli’ [14] and ‘Bartlett’ [15] are the assembled genomes, representing the Asian and European pears, respectively. When comparing the assembled quality of the two genomes (**Additional file 1: Table S1**), it is found that ‘Dangshansuli’ is a better choice for use as the reference genome due to the comparatively lower number of contigs N50 (6.6 Kb) and scaffolds N50 (88 Kb) observed for ‘Bartlett’. While contigs N50 and scaffold N50 are up to 35.7 Kb and 540.8 Kb for ‘Dangshansuli’, respectively. Furthermore, the anchoring ratio is far lower in ‘Bartlett’ (only 29.7%) than that for ‘Dangshansuli’ (75.5%). Based on longer assembled sequences at the chromosome level, we anticipate that most analyses, including linkage disequilibrium and sweep selection, will yield better results. Second, we performed synteny analysis for the two genomes. In ‘Bartlett’, a total of 991 scaffolds with 56,832 gene pairs and 7,512 synteny blocks with ‘Dangshansuli’ were identified with a length spanning 192.85 Mb, representing 33.4% of the ‘Bartlett’ genome. These results have revealed that there are large sequence differences between ‘Dangshansuli’ and ‘Bartlett’ genomes, and that they share a low homology. This suggests that we will lose some genetic information no matter which genome is used as a reference genome, particularly when both Asian and European populations are compared at the same time. Therefore, we have elected to choose the comparatively high-quality assembled ‘Dangshansuli’ genome as the reference genome. Thirdly, incidence of different mapping rates among samples has revealed differences of genetic components. In this study, mapping rates for pear accessions varied from a minimum of 33.9% to a maximum of 79.3%, with an average of 61.42%. This indicated that the selected germplasm of accessions used in this study represented the global diversity of pears. Differences in mapping rates between Asian and European pears indicated that higher levels of differences are present, which is consistent with results revealed by PCA, phylogenic tree, and population structure. In particular, the mapping rate of wild European pear accessions was the lowest based on the reference genome of ‘Dangshansuli’ (33.88%-35.80%). This is attributed to the far genetic relationship between wild European and cultivated Asian pears. Fourth, based on syntenic analysis, we have been able to find conserved regions between ‘Dangshansuli’ and ‘Bartlett’ genomes. This has allowed us to identify read coverage in these conserved regions. We have found that 13.42% region covered evenly by reads from both Asian and European accessions. In the downstream analysis, we attempted to use highly conserved regions with both missing rates of less than 10% in Asian and European pears, and our results have been consistent with our previous analysis (**Supplementary Table Q1**).

**Supplementary Table Q1.** Diversity of Asian and European pears.

| Missing rate | Group | SNP | CNS | π | SNP/Kb |
| --- | --- | --- | --- | --- | --- |
| < 0.5 | Asian | 14,590,066 | 164,642,842 | 4.92E-03 | 88.62 |
|  | European |  |  | 3.78E-03 |  |
| < 0.1 | Asian | 4,765,701 | 68,700,555 | 2.15E-03 | 69.37 |
|  | European |  |  | 1.51E-03 |  |

**NOTE 2**

**SNP variation and annotation in pear and in different groups**

A total of 18,302,883 SNPs were identified in pear with ~90 SNPs per kb (**Table 1**). This is a higher density than that found in peach [19] (20 SNPs per kb), poplar [70] (45 SNPs per kb), and date palm [22] (12 SNPs per kb). Furthermore, SNP densities in all 17 chromosomes of pear ranged between 8.65% to 9.3% (**Additional file 1: Table S9**).

Overall, a large difference in numbers of SNPs was detected between Asian (14.50 M) and European (6.94 M) pear groups (**Additional file 1: Table S2**). To account for differences in genome coverage, 164 M of commonly mapped genome regions in both Asian and European pears were analyzed for SNP content. It was revealed that the number of SNPs in Asian pears (13.38M) remained higher than that detected in European pears (6.42 M). Furthermore, 58.7% (4.30M) of SNPs found in the cultivated group (7.33M) were also identified in wild accessions of Asian pears, while 52.9% (1.88M) of SNPs found in the cultivated group (3.55M) were also detected in wild accessions of European pears. These results indicated that over half of the observed genetic vari­ations in cultivated pears were derived from wild pears, while all others were subsequently obtained during the process of domestication and natural adaptation. Of all identified SNPs in the pear genome, the majority (64.5%) were located in intergenic regions. Furthermore, 14.1% of pear SNPs were located in coding regions, which was higher than that found in peach [19] (10.0%), poplar [70] (5.6%), date palm [22] (6.1%), rice [23] (3.6%), soybean [17] (~3%), and maize [18] (~1%). This indicated that pear had higher levels of genetic variation than that found in annual crops, as well as in other trees studied thus far. The ratio of non-synonymous to synonymous substitutions in pear was 1.22, which was lower than that reported for peach [19] (1.31), poplar [70] (1.58), soybean [26] (1.36), and rice [23] (1.29), but higher than that of date palm [22] (1.17), maize [18] (1.14), sorghum [71] (1.00) and *Arabidopsis* [72] (0.83).

Among non-synonymous SNPs, those causing premature stops represented the largest group (59.36% or 18,376 genes) in pear, which was much higher than that reported for soybean [26] (46% or 3,018 genes). Presence of higher large-effect mutations suggested that the pear genome might have either accumulated a higher ratio of deleteri­ous mutations or instead got rid of disadvantageous mutations. Another explanation for the higher ratio of SNPs changing gene functions in pear might be due to recent whole genome duplication [14]. If most genes were present in two copies, then a mutation was less deleterious as the paralogue can maintain function. In addition, we investigated accumulation of large-effect changes in different pear groups, and found that a higher number of changes were present in the wild group than in the cultivated group, which might contribute to higher tolerance to wild environmental conditions.

**NOTE 3**

**Evolution rate of the *S-RNase* gene**

The evolution rate of *S-RNase* and other genes under balancing selection were calculated using the d/2T formula. Given that S-RNase-based gametophytic self-incompatibility is only found in eudicots which diverged less than 125 million years ago [73], we compared identities of *S-RNase* genes between *Pyrus* and *Solanum*; specifically, the *S-RNase* genes with the highest identity between *Pyrus* and *Solanum* were selected for calculation of d value. T value of *S-RNase* gene is less than 125 million years, because the *S-RNase* gene should be originated after the divergence of eudicots and monocots. So, the evolution rate of *S-RNase* gene is underestimated.

For the compared balancing selection genes, it is difficult to evaluate the evolution rate of genes after divergence of *Pyrus* and *Solanum*. So, we identified the balancing selection genes originated earlier than *S-RNase* genes through comparing pear with ancient species, *Selaginella moellendorffii* diverged at ~ 400 MYA [74]. The alignment conducted using Muscle was filtered with the following three criteria: 1) coverage for a gene in pear was over 60%; 2) if the coverage for a gene in pear was greater than 90%, the alignment was used directly; 3) if the coverage for a gene in pear was less than 90%, the alignment with greats map region was used. According to the three criteria, the sequence identity of balancing selection gene were determined and the corresponding d values were calculated; while the T value used divergence time ~ 400 MYA, which might less than the actual evolution time of genes. Thus, the underestimated evolution rate of *S-RNase* gene compared with the overestimated evolution rates of the detected balancing selection genes, the results still revealed higher evolution rate of *S-RNase* gene (1.91e-09 sites/year) than the other balancing selection genes (ranged between 2.31e-10 and 6.10e-10).

70. Evans LM, Slavov GT, Rodgers-Melnick E, Martin J, Ranjan P, Muchero W, et al. Population genomics of *Populus trichocarpa* identifies signatures of selection and adaptive trait associations. Nat Genet. 2014;46:1089-1096.

71. Mace ES, Tai S, Gilding EK, Li Y, Prentis PJ, Bian L, et al. Whole-genome sequencing reveals untapped genetic potential in Africa's indigenous cereal crop sorghum. Nat Commun. 2013;4:2320.

72. Clark RM, Schweikert G, Toomajian C, Ossowski S, Zeller G, Shinn P, et al. Common sequence polymorphisms shaping genetic diversity in *Arabidopsis thaliana*. Science. 2007;317:338-342.

73. Bell CD, Soltis DE, Soltis PS. The age and diversification of the angiosperms re-revisited. Am J Bot. 2010;97:1296-1303.

74. Weng J, Tanurdzic M, Chapple C. Functional analysis and comparative genomics of expressed sequence tags from the lycophyte *Selaginella moellendorffii*. BMC Genomics. 2005;6:85.

**Supplementary Tables**

**Table S1. Comparisons of Asian pear genome of ‘Dangshansuli’ and European genome of ‘Bartlett’.**

| **Contigs** | **‘Bartlett’** | **‘Dangshansuli’** |
| --- | --- | --- |
| Number of contigs | 182,196 | 25,312 |
| Total size of contigs (Mb) | 507.7 | 501.3 |
| N50 contig length (Kb) | 6.6 | 35.7 |
| Longest contig (Mb) | 0.1 | 0.3 |
| **Scaffolds** |  |  |
| Number of scaffolds | 142083 | 2,103 |
| Total size of scaffolds (bp) | 577 | 512 |
| N50 scaffold length (Kb) | 88.1 | 540.8 |
| Longest scaffold (Mb)  Anchored size to the chromosome (Mb)  Anchored rate to the chromosome (%) | 1.2  171.3  29.7 | 4.1  386.7  75.5 |

**Table S2. Summary of SNP distribution in genome for all samples and different groups.**

| **Groups**  **(Number of samples)** | **Total number** | **Synonymous** | **Non-Synonymous** | **Non-Syn/Syn** | **mRNA** | **UTR** | **CDS** | **Intron** | **Intergenic** |
| --- | --- | --- | --- | --- | --- | --- | --- | --- | --- |
| All (113) | 18,302,883 | 1,160,664 | 1,416,121 | 1.22 | 6,494,008 | 460,862 | 2,576,785 | 3,456,361 | 11,808,875 |
| Asian(63) | 14,501,253 | 816,279 | 969,426 | 1.19 | 4,606,134 | 333,918 | 1,785,705 | 2,486,511 | 9,895,119 |
| European(50) | 6,945,796 | 584,039 | 679,346 | 1.16 | 2,936,311 | 191372 | 1,263,385 | 1,481,554 | 4,009,485 |
| Cultivated(56) | 10,902,511 | 661,128 | 763,792 | 1.16 | 3,646,406 | 362,275 | 1,424,920 | 1,959,156 | 7,256,105 |
| Wild(57) | 13,540,936 | 991,570 | 1,171,683 | 1.18 | 5,287,665 | 189,235 | 2,163,253 | 2,762,137 | 8,253,271 |
| Wild Asian (32) | 10,510,280 | 663,695 | 764,122 | 1.15 | 3,603,265 | 255,206 | 1,427,817 | 1,920,242 | 6,907,015 |
| Cultivated Asian (31) | 8,441,743 | 487,956 | 550,286 | 1.13 | 2,645,724 | 189,235 | 1,038,242 | 1,418,247 | 5,796,019 |
| Wild European (25) | 4,894,247 | 468,731 | 534,506 | 1.14 | 2,257,062 | 139,953 | 1,003,237 | 1,113,872 | 2,637,185 |
| Cultivated European (25) | 4,220,232 | 322,949 | 358,988 | 1.11 | 1,632,956 | 110,219 | 681,937 | 840,800 | 2,587,276 |

**Table S3. Summary of Large effect SNPs in genomes of all samples and in different groups.**

| **Groups** | **Total number** | | **Premature stop** | **Stop codon to non-stop codon** | **Start codon to non-start codon** | **Splice sites** | **Number of involved genes** |
| --- | --- | --- | --- | --- | --- | --- | --- |
| All (113) | 30,959 | 18,376 | | 1,964 | 2,078 | 8,541 | 13,858 |
| Asian(63) | 22,812 | 13,603 | | 1,582 | 1,613 | 6,014 | 11,636 |
| European(50) | 14,439 | 8,997 | | 926 | 936 | 3,580 | 8,612 |
| Asian wild (32) | 17,562 | 10,486 | | 1,271 | 1,249 | 4,556 | 9,810 |
| Asian cultivated (31) | 12,711 | 7,520 | | 1,073 | 947 | 3,171 | 8,046 |
| European wild (25) | 10,983 | 6,905 | | 692 | 707 | 2,679 | 7,112 |
| European cultivated (25) | 7,790 | 4,814 | | 553 | 515 | 1,908 | 5,532 |

**Table S4. LD decays in different pear groups.**

| **Pear groups** | **SNP Group** | | |
| --- | --- | --- | --- |
|  | **r^2^** | **Half distance (bp)** | **Segment** |
| Cultivated Asian | 0.4424 | 553 | 800 |
| Wild Asian | 0.3210 | 83 | 200 |
| Cultivated European | 0.4239 | 154 | 400 |
| Wild European | 0.3564 | 55 | 0 |

**Table S5. List of samples from subgroup 1 and subgroup2 of European pear - Group I.**

| **Subgroup 1** | | **Subgroup 2** | |
| --- | --- | --- | --- |
| Sample | Region | Sample | Region |
| Pyw_re1  Pyw_re2  Pyw_sy1  Pyw_sac1  Pyw_sa2  Pyw_sa1  Pyw_el2  Pyw_sy2  Pyw_gl1  Pyw_el1 | America  Kazakhstan  Iran  Georgia  Georgia  England  Armenia  Israel  Turkey  Turkey | Pyw_sp1  Pyw_sp2  Pyw_cos1  Pyw_cos2  Pyw_ni2  Pyw_co1  Pyw_kor1  Pyw_cau1  Pyw_cau2  Pyw_co2  Pyw_pyr1  Pyw_pyr2  Pyw_ma1  Pyw_ma2 | Yugoslavia  Sardegna  Netherlands  Russia  America  England  Kirgizstan  Armenia  Russia  France  Hungary  Iran  England  Morocco |
|  | |  | |

**Table S6. The diversity of the *S-RNas*e gene for different groups of Asian and European pears.**

| **Groups** | **SNP number** | **Sum of ϴw** | **Sum of ϴπ** | **Common coverage(bp)** | **ϴw** |
| --- | --- | --- | --- | --- | --- |
| Asian cultivated | 121 | 43.83 | 19.80 | 409 | 1.07E-01 |
| Asian wild | 135 | 44.01 | 20.27 | 409 | 1.08E-01 |
| European cultivated | 92 | 48.50 | 18.67 | 445 | 1.09E-01 |
| European wild | 141 | 48.62 | 24.09 | 445 | 1.09E-01 |

**Table S7. Confirmation of *S-RNase* alleles domesticated by balancing selection.**

|  | **Asian** | | **European** | |
| --- | --- | --- | --- | --- |
|  | Cultivated | Wild | Cultivated | Wild |
| Pi | 0.169 | 0.172 | 0.160 | 0.171 |
| Tajima’s D | 2.35 | 2.08 | 2.07 | 2.34 |
| Fst | 0.0159 | | 0.0325 | |

**Table S8. Admixture samples in different pear groups.**

|  | **Cultivated** |  | **Wild** |  | **Cultivated** | **Wild** |
| --- | --- | --- | --- | --- | --- | --- |
| **Asian**  **Admixture** | | Pyc_si1 | | --- | | Pyc_si2 | | Pyc_si3 | | Pyc_si4 | |  | Pyw_ar1 | **European**  **Admixture** | Pyc_co6 | Pyw_ni1 |
|  |  |  | Pyw_ho1  Pyw_ho2 |  |  |  |
|  |  |  | Pyw_ph1  Pyw_ph2  Pyw_ph3 |  |  |  |
|  |  |  | Pyw_xe1  Pyw_xe2 |  |  |  |

**Table S9. Distribution of all SNPs in different pear chromosomes.**

| **Chromosome** | **SNP** | **Effective Site** | **Chromosome Length** | **SNP Density** | **SNP Rate** |
| --- | --- | --- | --- | --- | --- |
| Chr01 | 509,927 | 5,821,038 | 10,691,755 | 8.76% | 2.79% |
| Chr02 | 1,061,304 | 11,642,853 | 22,098,781 | 9.12% | 5.80% |
| Chr03 | 1,198,072 | 13,058,876 | 27,392,285 | 9.17% | 6.55% |
| Chr04 | 558,232 | 6,085,246 | 13,384,095 | 9.17% | 3.05% |
| Chr05 | 1,330,977 | 14,400,393 | 28,442,882 | 9.24% | 7.27% |
| Chr06 | 913,543 | 10,133,094 | 23,112,003 | 9.02% | 4.99% |
| Chr07 | 643,363 | 7,021,580 | 15,267,112 | 9.16% | 3.52% |
| Chr08 | 776,335 | 8,374,554 | 17,110,699 | 9.27% | 4.24% |
| Chr09 | 1,015,654 | 11,203,502 | 22,428,363 | 9.07% | 5.55% |
| Chr10 | 1,043,920 | 11,597,948 | 26,220,497 | 9.00% | 5.70% |
| Chr11 | 1,086,492 | 12,176,991 | 30,316,187 | 8.92% | 5.94% |
| Chr12 | 959,943 | 10,322,800 | 22,757,174 | 9.30% | 5.24% |
| Chr13 | 756,227 | 8,510,634 | 15,147,870 | 8.89% | 4.13% |
| Chr14 | 819,285 | 9,152,699 | 20,263,496 | 8.95% | 4.48% |
| Chr15 | 1,830,272 | 20,794,205 | 43,574,056 | 8.80% | 10.00% |
| Chr16 | 833,336 | 9,153,527 | 20,649,150 | 9.10% | 4.55% |
| Chr17 | 983,595 | 10,676,421 | 25,332,008 | 9.21% | 5.37% |
| unanchored | 1,982,406 | 22,916,512 | 124,578,682 | 8.65% | 10.83% |
| Sum | 18,302,883 | 203,042,873 | 508,767,095 |  |  |

**SNP Rate= Chr SNP number/Total SNP number**

**SNP Density = Chr SNP number/Effective Site number**

**Supplementary Figures**

**Fig. S1. World-wide collections of pear accessions used in this study.**

To represent as much genetic diversity of pear germplasm as possible, we selected 63 Asian accessions including 31 cultivated and 32 wild pears genotypes, and 50 European accessions including 25 cultivated and 25 wild genotypes, originated in wide geographic regions and in different countries. These are denoted by colored dots for different groups on the world map.


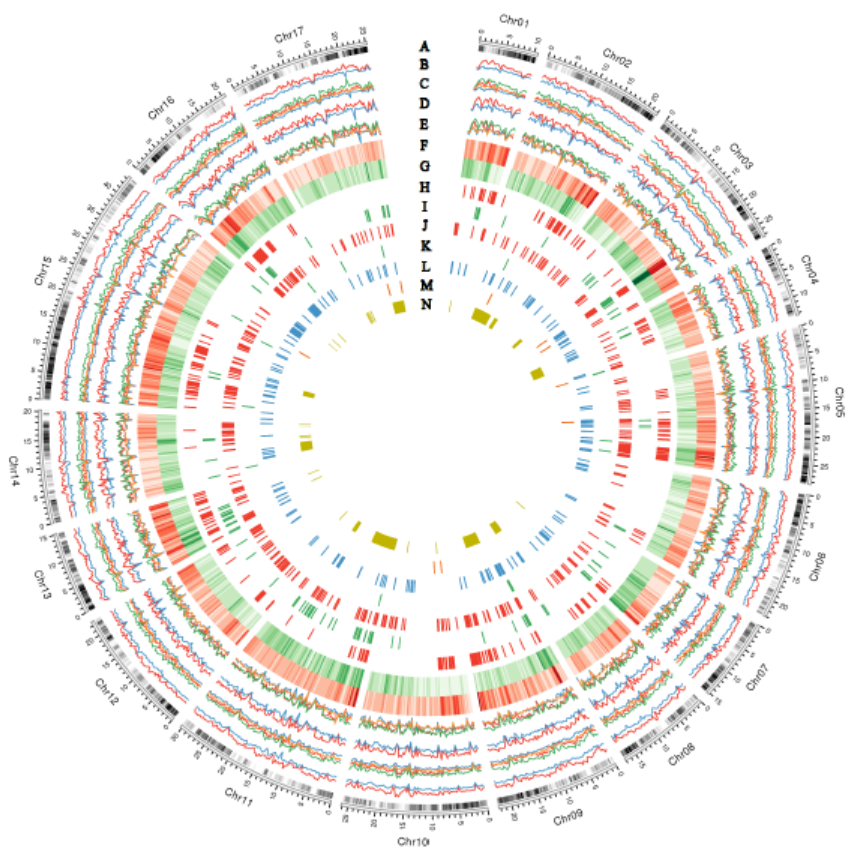


**Fig. S2. Summary of pear whole genome re-sequencing data.**

Concentric circles demonstrate different sequencing features using the Circos program. All 17 chromosomes of pear are portrayed along the perimeter of each circle. (A) Distribution of gene density (300 kb window); (B) Distribution of SNP density in Asian (red) and European (blue) pear groups; (C) Distribution of SNP density in cultivated (red) and wild (green) Asian pears, and in cultivated (blue) and wild (orange) European pear groups; (D) Distribution of ϴ_π_ in Asian (red) and European (blue) pear groups; (E) Distribution of ϴ_π_ in cultivated (red) and wild (green) Asian pears, and in cultivated (blue) and wild (orange) European pears; (F) Fst values of wild compared with cultivated Asian pears; (G) Fst values of wild compared with cultivated European pears; (H) Selection region during domestication from wild to cultivated Asian pears; (I) Selection region during domestication from wild to cultivated European pears; (J) Block distribution in cultivated Asian pears; (K) Block distribution in wild Asian pears; (L) Block distribution in cultivated European pears; (M) Block distribution in wild European pears.

**Fig. S3. Population structure (K= 2-7) of all 113 pear accessions.**

(a) Each color corresponds to a single population as noted. Each accession is represented by a vertical bar. The Y-axis refers to the proportion of the genetic background, and the height of each line with different colors represents the probability of an accession belonging to a different genetic background. (b) Delta K showed a peak at 2, suggesting two clusters as the most appropriate option, which supports the phylogenic tree and PCA result of “two major discrete clusters of Asian and European groups were detected”. When K was 3-7, for the Asian group, pear accessions clustered into four subgroups, and there was a little change in European pears. The phylogenic tree’s branches had coincidence with the structure results in Figure 2a.


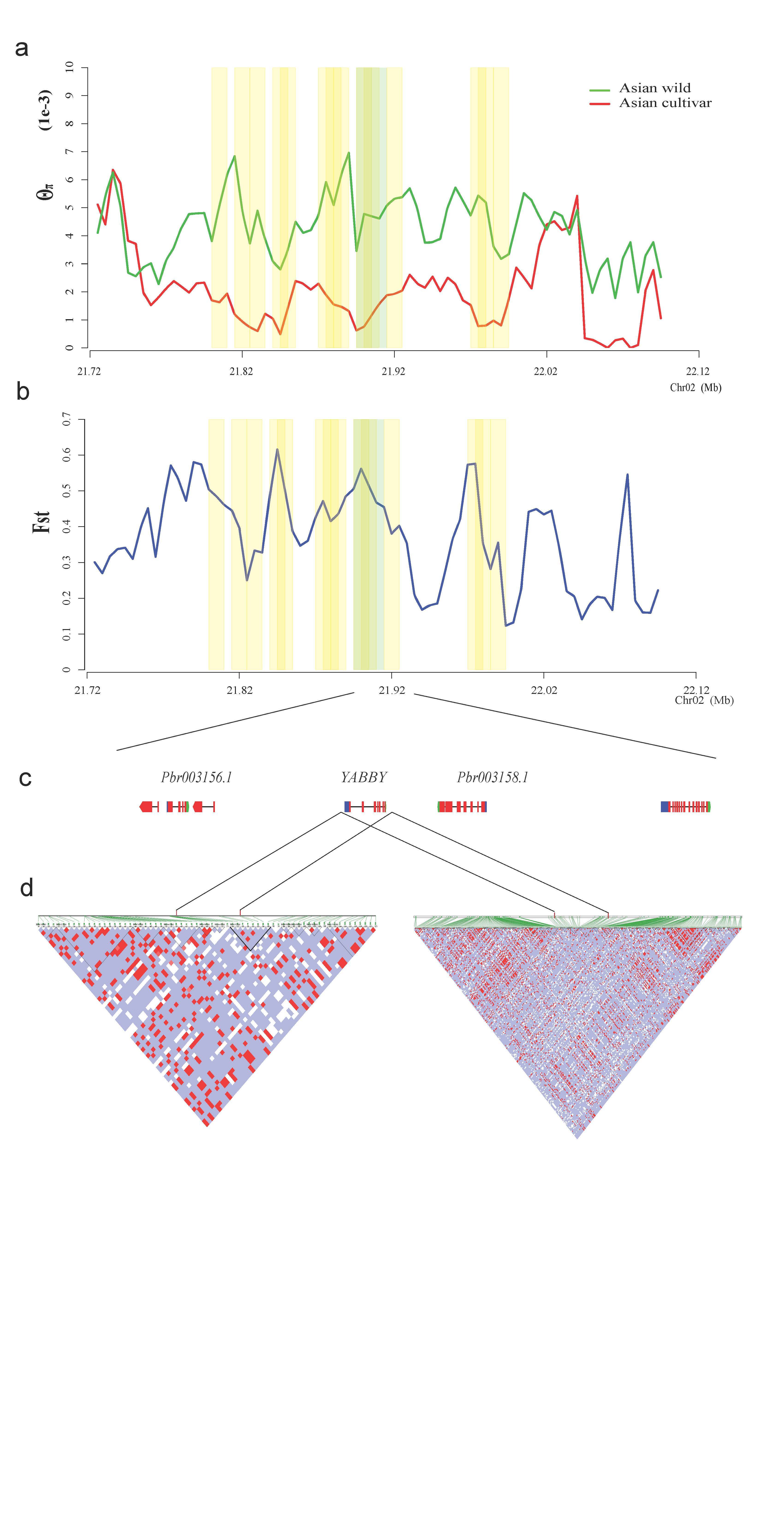


**Fig. S4. Fruit size-related genes in selective sweeps of Asian pears**

The YABBY gene related to fruit size. (a) Differences in ϴπ values between wild and cultivated Asian groups; (b) FST values between wild and cultivated Asian groups in 10-kb intervals; (c) Predicted genes within domesticated regions in cultivated and wild Asian groups; (d) LD blocks around candidate genes under selection. Red and white spots indicate strong (r2=1) and weak (r2=0) correlations, respectively.


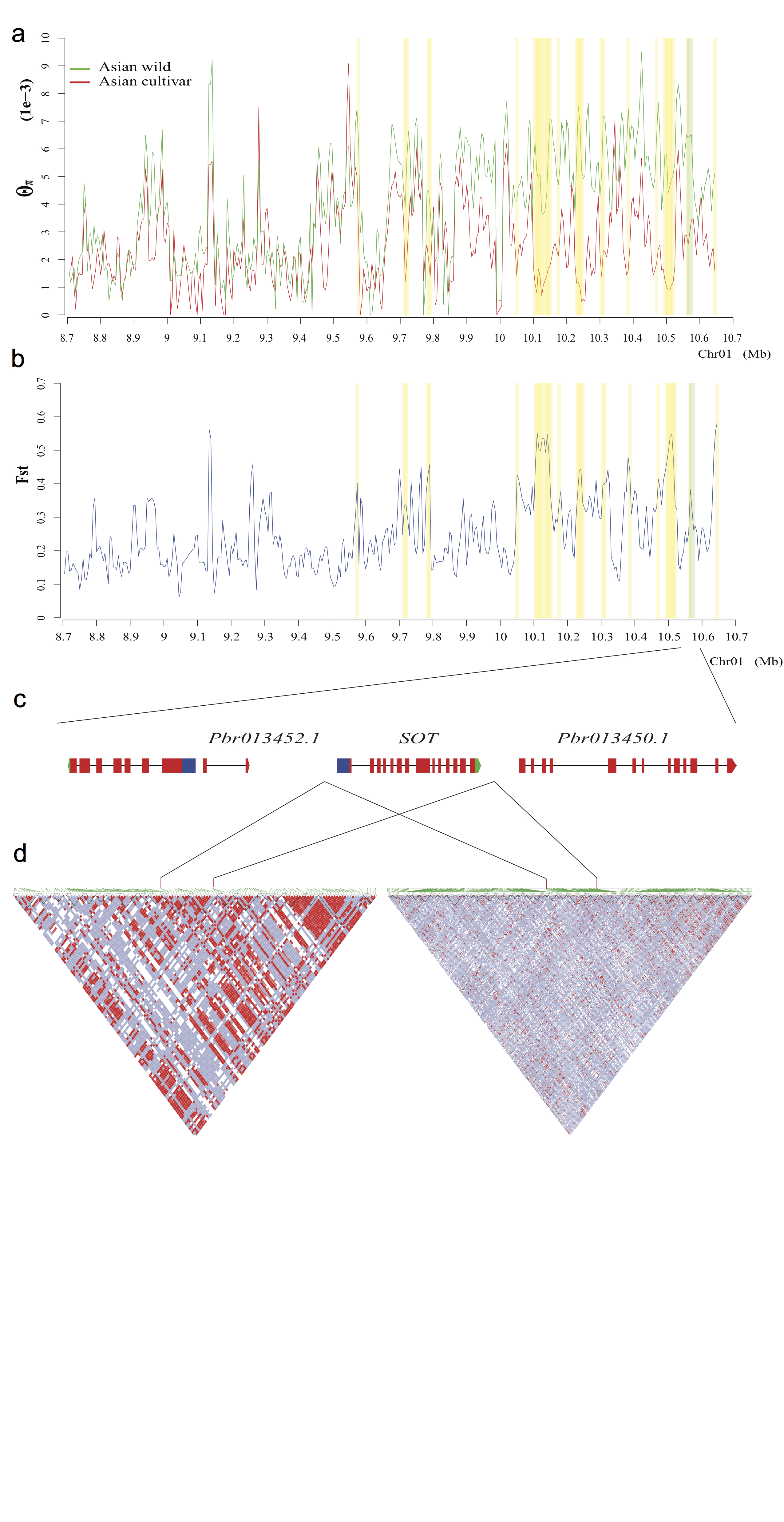


**Fig. S5. Sugar-related genes in selective sweeps of Asian pears**

The SOT gene related to sugar content of fruit; 3B. The YABBY gene related to fruit size. (a) Differences in ϴπ values between wild and cultivated Asian groups; (b) FST values between wild and cultivated Asian groups in 10-kb intervals; (c) Predicted genes within domesticated regions in cultivated and wild Asian groups; (d) LD blocks around candidate genes under selection. Red and white spots indicate strong (r2=1) and weak (r2=0) correlations, respectively.


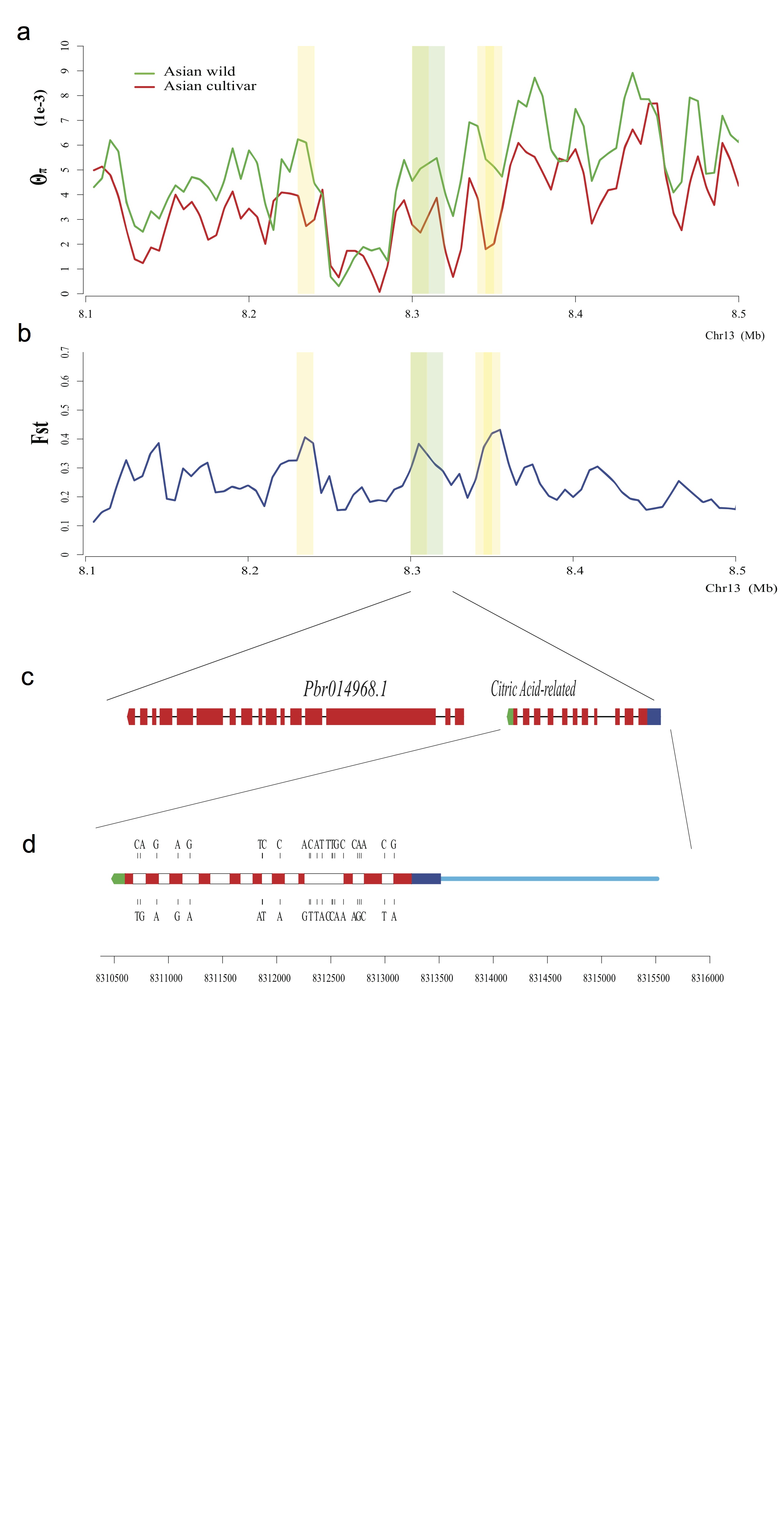


**Fig. S6. Acid-related genes in selective sweeps of Asian pears.**

(a) Differences in ϴ_π_ values between wild and cultivated groups; (b) FST values between wild and cultivated groups in 10-kb intervals; (c) Predicted genes within domesticated regions in cultivated and wild groups; (d) Predicted gene models and SNPs loci in genomic and promoter regions. Cyan line denotes upstream 2 Kb region, red square denotes CDS regions, white square denotes intron regions, blue line denotes UTR_5', and green line denotes UTR_3'.

**
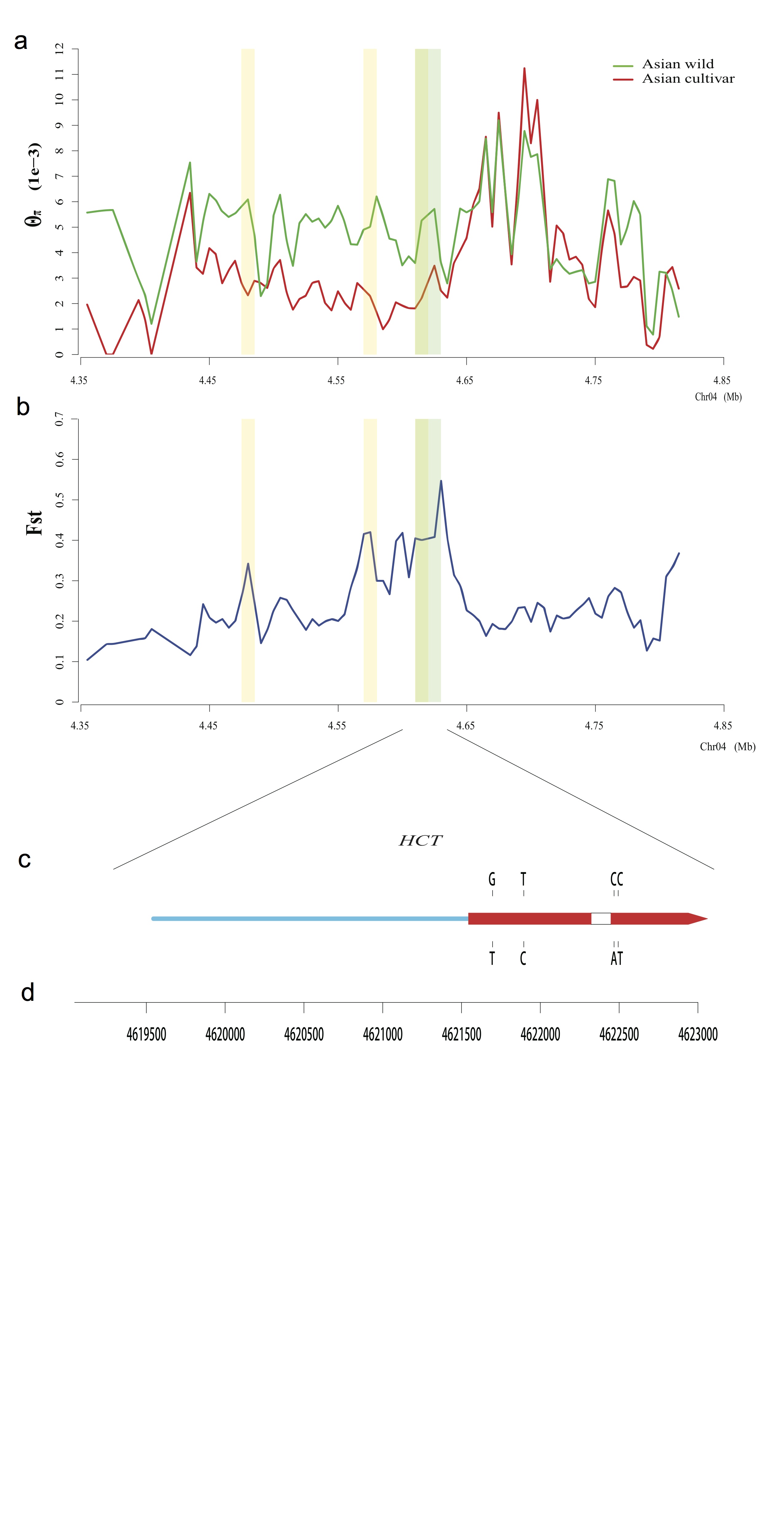

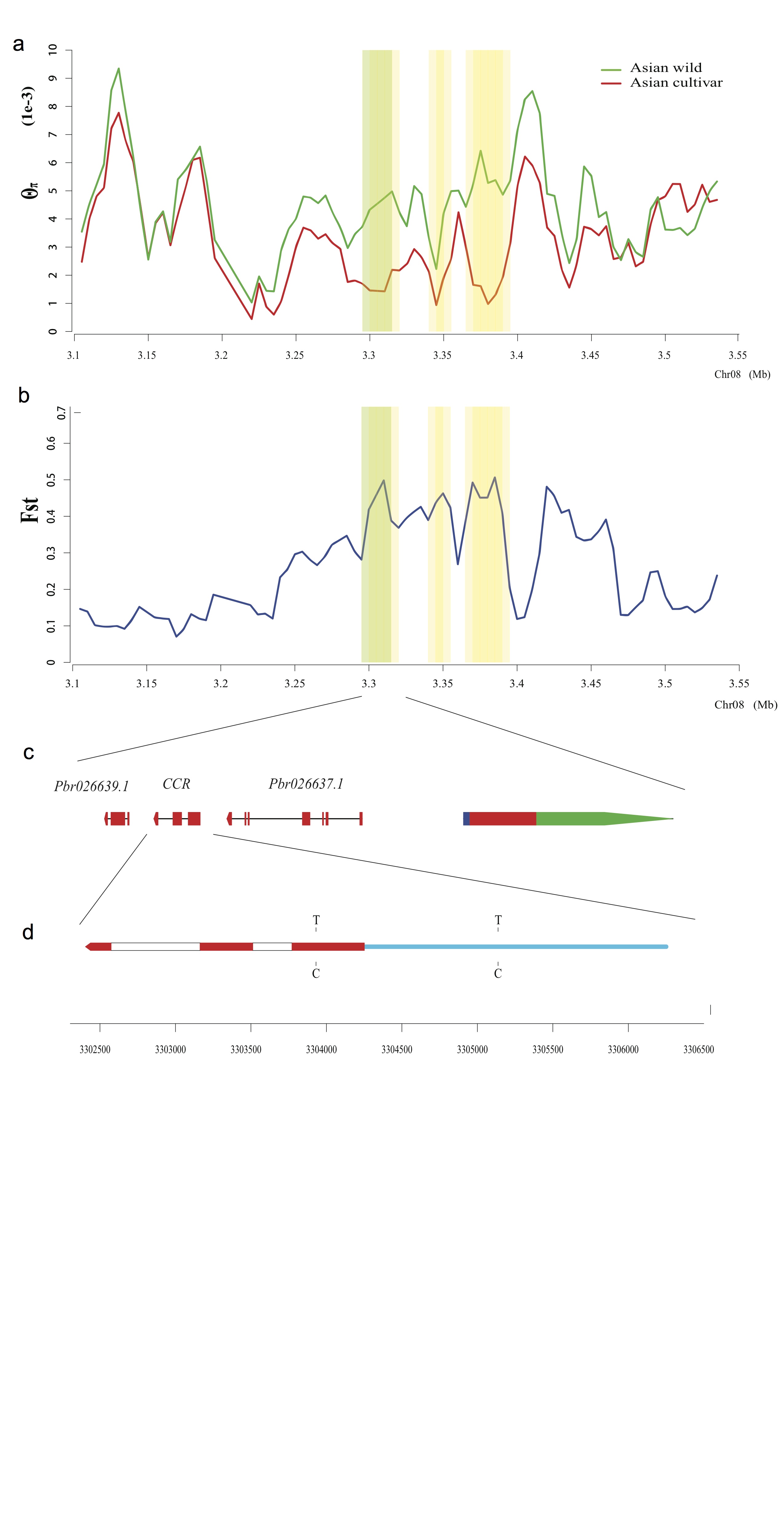
**

**
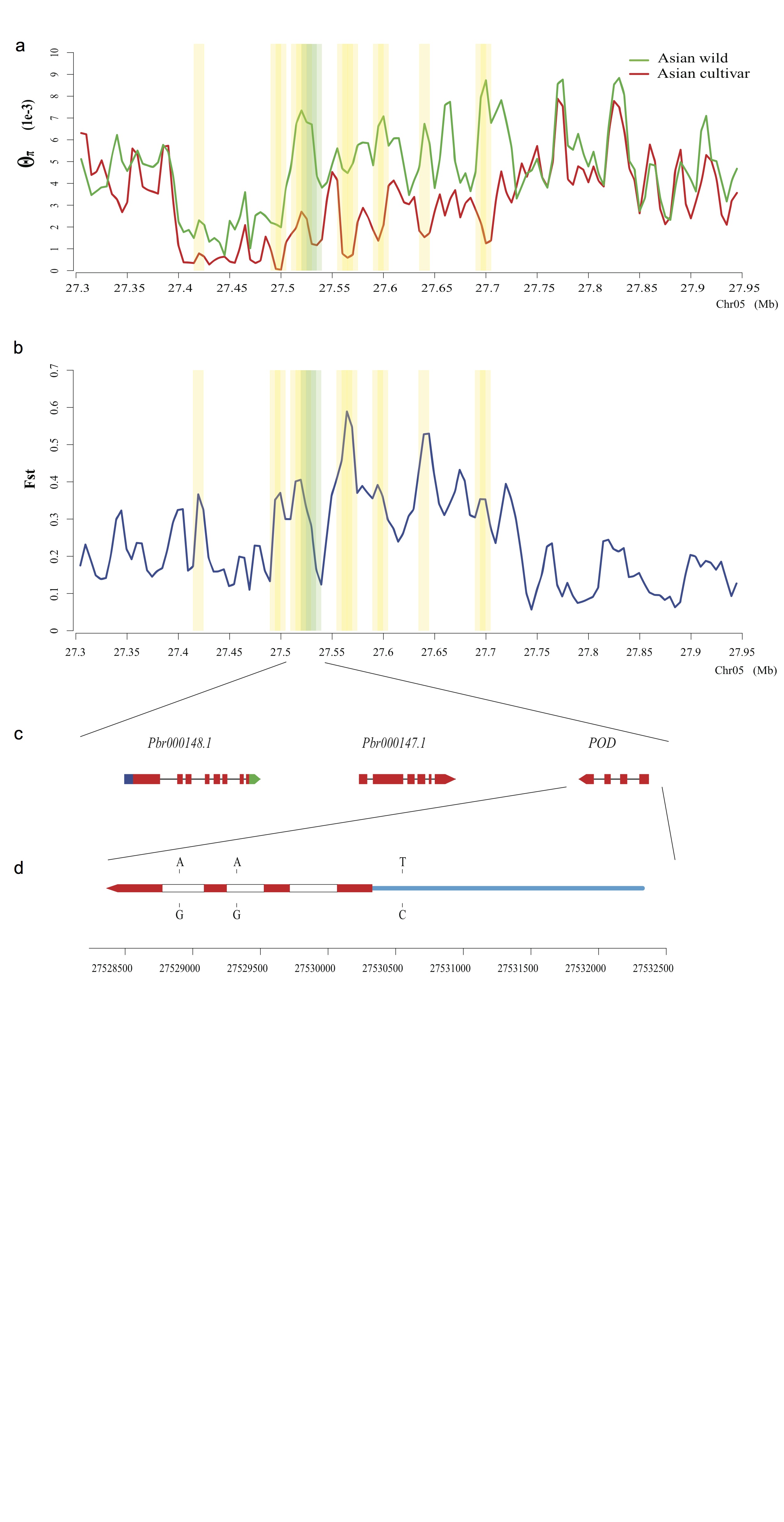

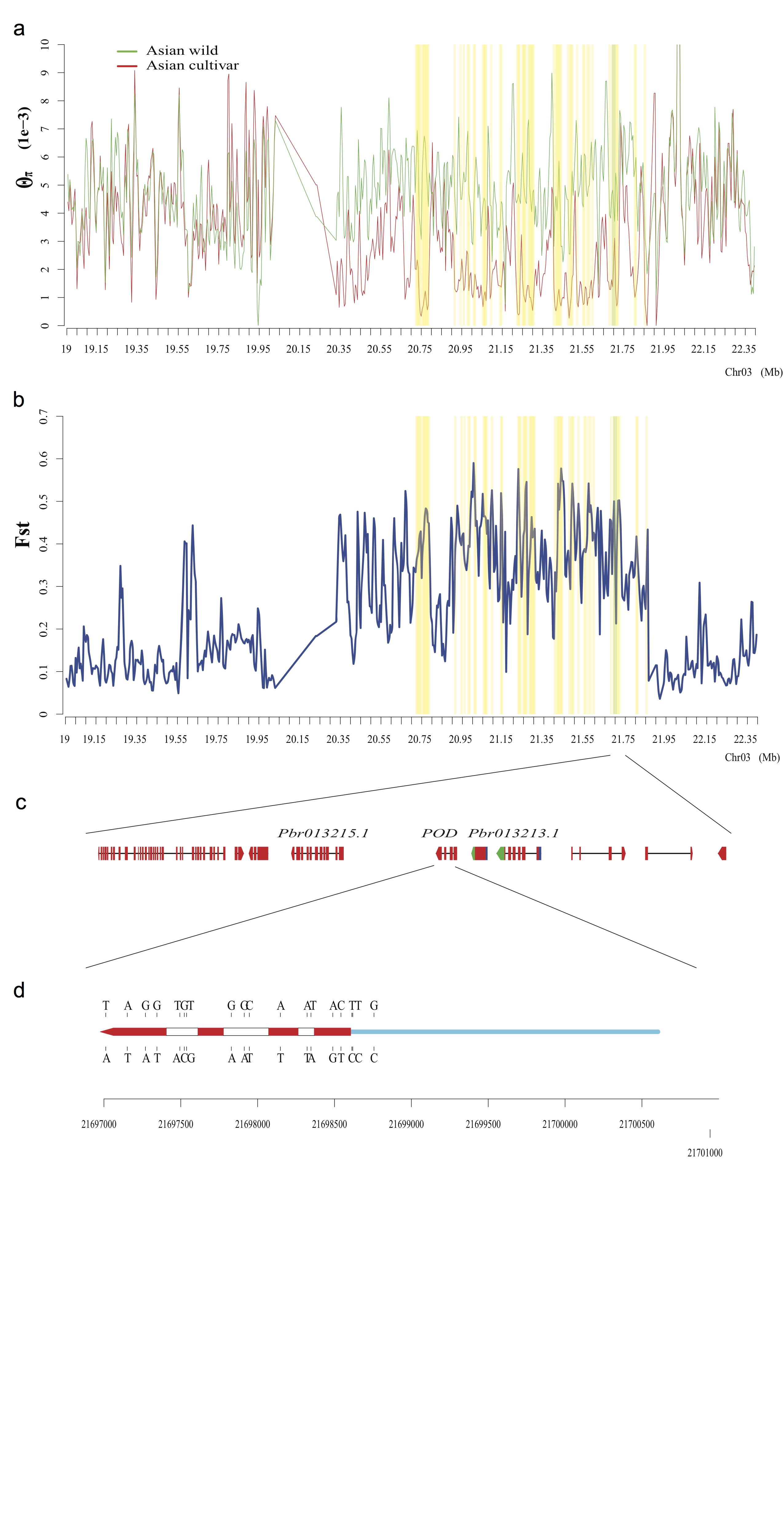
**

**Fig. S7. Stone cell-related genes in selective sweeps of Asian pears.**

(a) Differences in ϴ_π_ values between wild and cultivated group; (b) FST values between wild and cultivated groups in 10-kb intervals; (c) Predicted genes within domesticated regions in cultivated and wild groups; (d) Predicted gene models and SNPs loci in genomic and promoter regions. Cyan line denotes upstream 2 Kb region, red square denotes CDS regions, white square denotes intron regions, blue line denotes UTR_5', and green line denotes UTR_3'.
